# Future Developments in Charged Particle Therapy: Improving Beam Delivery for Efficiency and Efficacy

**DOI:** 10.3389/fonc.2021.780025

**Published:** 2021-12-09

**Authors:** Jacinta Yap, Andrea De Franco, Suzie Sheehy

**Affiliations:** ^1^ School of Physics, University of Melbourne, Melbourne, VIC, Australia; ^2^ IFMIF Accelerator Development Group, Rokkasho Fusion Institute, National Institutes for Quantum Science and Technology, Aomori, Japan

**Keywords:** particle therapy, accelerators, large energy acceptance, energy layer switching time, rescanning, FLASH, arc therapy, beam delivery

## Abstract

The physical and clinical benefits of charged particle therapy (CPT) are well recognized. However, the availability of CPT and complete exploitation of dosimetric advantages are still limited by high facility costs and technological challenges. There are extensive ongoing efforts to improve upon these, which will lead to greater accessibility, superior delivery, and therefore better treatment outcomes. Yet, the issue of cost remains a primary hurdle as utility of CPT is largely driven by the affordability, complexity and performance of current technology. Modern delivery techniques are necessary but limited by extended treatment times. Several of these aspects can be addressed by developments in the beam delivery system (BDS) which determines the overall shaping and timing capabilities enabling high quality treatments. The energy layer switching time (ELST) is a limiting constraint of the BDS and a determinant of the beam delivery time (BDT), along with the accelerator and other factors. This review evaluates the delivery process in detail, presenting the limitations and developments for the BDS and related accelerator technology, toward decreasing the BDT. As extended BDT impacts motion and has dosimetric implications for treatment, we discuss avenues to minimize the ELST and overview the clinical benefits and feasibility of a large energy acceptance BDS. These developments support the possibility of advanced modalities and faster delivery for a greater range of treatment indications which could also further reduce costs. Further work to realize methodologies such as volumetric rescanning, FLASH, arc, multi-ion and online image guided therapies are discussed. In this review we examine how increased treatment efficiency and efficacy could be achieved with improvements in beam delivery and how this could lead to faster and higher quality treatments for the future of CPT.

## 1 Introduction

Access to charged particle therapy is growing rapidly worldwide. As a therapeutic modality CPT is now well established, where proton beam therapy (PBT) is the most common type. Where available, PBT is often standard practice, particularly for pediatric cases and specific tumor types [ocular, head and neck tumors (1)]. CPT has an important prospective role in reducing the growing cancer burden on a global scale, and its impact could be significant (2) however, its full potential is yet to be realized. Overcoming this requires improvements in two key areas: improving efficacy and decreasing cost.

CPT offers benefits over and above standard radiotherapy (RT) for palliative or curative treatments, offering not just physical dose escalation but also biological advantage. Yet in terms of efficacy, we cannot capitalize fully on increased radiobiological effectiveness of charged particles at present, primarily due to limitations in knowledge (3). For this reason, existing programmes of research are investigating the underlying mechanisms of CPT in terms of fundamental chemical, biological and cellular processes (4–9) to try to understand the roles of these processes in determining clinical outcomes (10–12). Nonetheless, the superior physical properties of charged particles are evident as the characteristic ‘*Bragg Peak*’ (BP) enables a precise dose distribution and an improved therapeutic window.

The second reason we cannot yet fully exploit the efficacy of CPT is due to technological limitations. Advanced techniques and technological improvements for CPT seek to deliver higher quality treatments with increased conformity as these translate to long-term benefits. However some of these improvements would increase, rather than decrease, the cost of the treatment.

In terms of cost (or efficiency) the gap between conventional X-ray photon RT (XRT) and CPT still exists due to the many challenges to be addressed: affordability, complexity and limitations with current technology all restrict the utility of CPT. Developments in accelerators and related technologies, beam delivery methods, verification tools, and increased clinical experience have seen growth in the number of facilities. Although availability has surged in recent years with several vendors offering competitive and commercial turn-key solutions, high capital and operational costs are still a primary issue. Many potential areas of improvement have been well identified (13–21). In general, the potential for cost reduction can be considered by decreasing the facility and machine size, operational complexities, treatment times, increasing the treatment workflow efficiency and hence throughput.

However, simply making the treatment cheap and widespread is not enough. Both efficiency and efficacy need to be improved, in other words, even in an ideal world of low-cost and widespread availability of facilities, the maximum possible clinical benefit of CPT will only be achievable if existing technical limitations are overcome. Of course, there are many improvements which can be implemented with current systems to improve treatment efficacy and cost. Yet these will be restricted by – both technological and systems-based – capabilities of the BDS: developments are necessary to better deliver CPT, at future facilities and with advanced methodologies.

Presently, the majority of CPT treatments use active pencil beam scanning (PBS), involving the intricate delivery of several thousand overlapping narrow beams, resulting in highly conformal dose distributions. Most clinical indications are treated with PBS however the lengthy beam delivery time is consequential. During treatment the beam is scanned across the target site, requiring the accelerator complex, control system and diagnostic instruments to adjust. A key bottleneck in this process is the energy layer switching time.

The slow ELST is due to technical constraints and is a prevalent issue: it can account for a majority of the BDT (22, 23). The beam moves relatively quickly across the tumor transversely, but often takes much longer to switch the beam in depth. This extends the beam-on time and long overall irradiation times can increase dose uncertainties. This is a key problem for cases where the tumor site itself may also move, for example during lung treatments, interplay effects caused by respiratory motion are unavoidable. Although different motion management approaches such as gating, rescanning and tracking can be performed, the clinical implications reduce the utility of CPT, especially for particular indications (11). Moreover, as the high dose BP region is a motivation for using CPT, sensitivity to changes in range are especially important – for heavy ions this is even greater – which impairs benefits attained from the physical and radiobiological advantages (24, 25).

There are many factors which govern the overall cost and efficacy of treatment and we identify one key underlying aspect: the beam delivery. Complimentary to the accelerator complex, the beam delivery system contributes significantly to the BDT and thus overall treatment time, forming the primary focus of this review. The scope of this review is to look at the technology pertaining to the BDS, broadly covering those aspects of the system which impact the dose delivery process and treatment time^1^. We review the challenges and developments needed for the BDS and related accelerator technology with the outlook of decreasing the BDT. The clinical feasibility, impact on the delivery process and potential benefits of different approaches are examined. We focus on the perspective of minimizing the ELST, its contribution and the implication of extended BDT on treatment. Lastly, we examine aspects to deliver emerging and future treatment paradigms such as FLASH, arc, multi-ion and image guided therapy.

## 2 Beam Delivery

**Figure 1:**
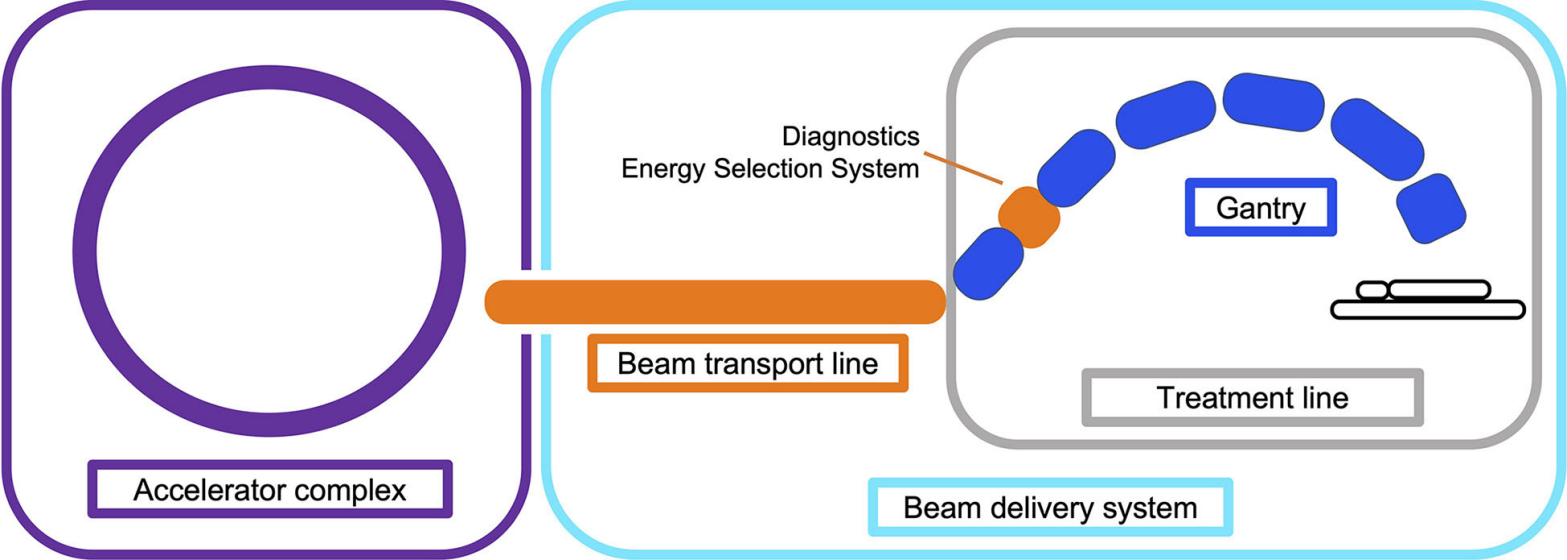
Illustration overviewing the main components in a CPT treatment facility. The BDS is shown to include the BTL (beam instrumentation and devices which may alter the energy or shape of the beam) and treatment line (fixed line or here, a gantry).

The beam produced by the accelerator must be shaped and modified to deliver the dose to the target site. This involves changing the distribution of the spatial (lateral) and energy (longitudinal) spread and also often the time structure, i.e. beam modification in four-dimensions. The objective of the BDS is to deliver this beam with the required parameters prescribed by the treatment plan. In this review we define the BDS as the components after the accelerator complex which determines how the beam is shaped, transported and ultimately delivered to the patient for treatment. This encompasses the beam transport lines (BTL), diagnostic instrumentation, energy selection systems (ESS), treatment line (gantry or fixed delivery line) and treatment head. For brevity, we focus mainly on the BTL, treatment line and delivery system for PBS (**Figure 1**).

Treatments can take anywhere from a few minutes to more than half an hour, depending on the tumor type, size and complexity of the plan. This is determined by the BDT, additional activities including patient set-up (immobilisation, imaging, unload etc.) and equipment related checks (couch positioning, beam checks, readying beam devices etc.). The BDT consists of the actual irradiation ‘*beam-on*’ time and the ‘*beam-off*’ time, spent requesting and waiting on the beam after adjustments or between fields.

A quantitative analysis of PBT treatments by Suzuki et al. (26) reported that approximately 80% of treatment time was spent on these additional activities with the remaining 20% contributed by the BDT. Total treatment time is shown to increase with the number of fields; complex cases required the same amount of time to carry out patient-related activities but accrued larger contributions from equipment checks and the BDT. Reductions in these latter aspects have more potential to improve the efficiency as patient-related process times can vary widely, depend on the physical and clinical condition of the patient and cannot necessarily be improved with technology. Furthermore, shorter treatment times are preferred not just for cost but also due to difficulty of immobilisation and set-up of patients for 30 minutes or longer. As discussed by Nystrom & Paganetti et al. (13), a faster BDT can result in a significant gain in treatment efficiency, particularly for multi-room facilities with high waiting times. Evidently, any increase in treatment efficiency is valuable.

**Figure 2:**
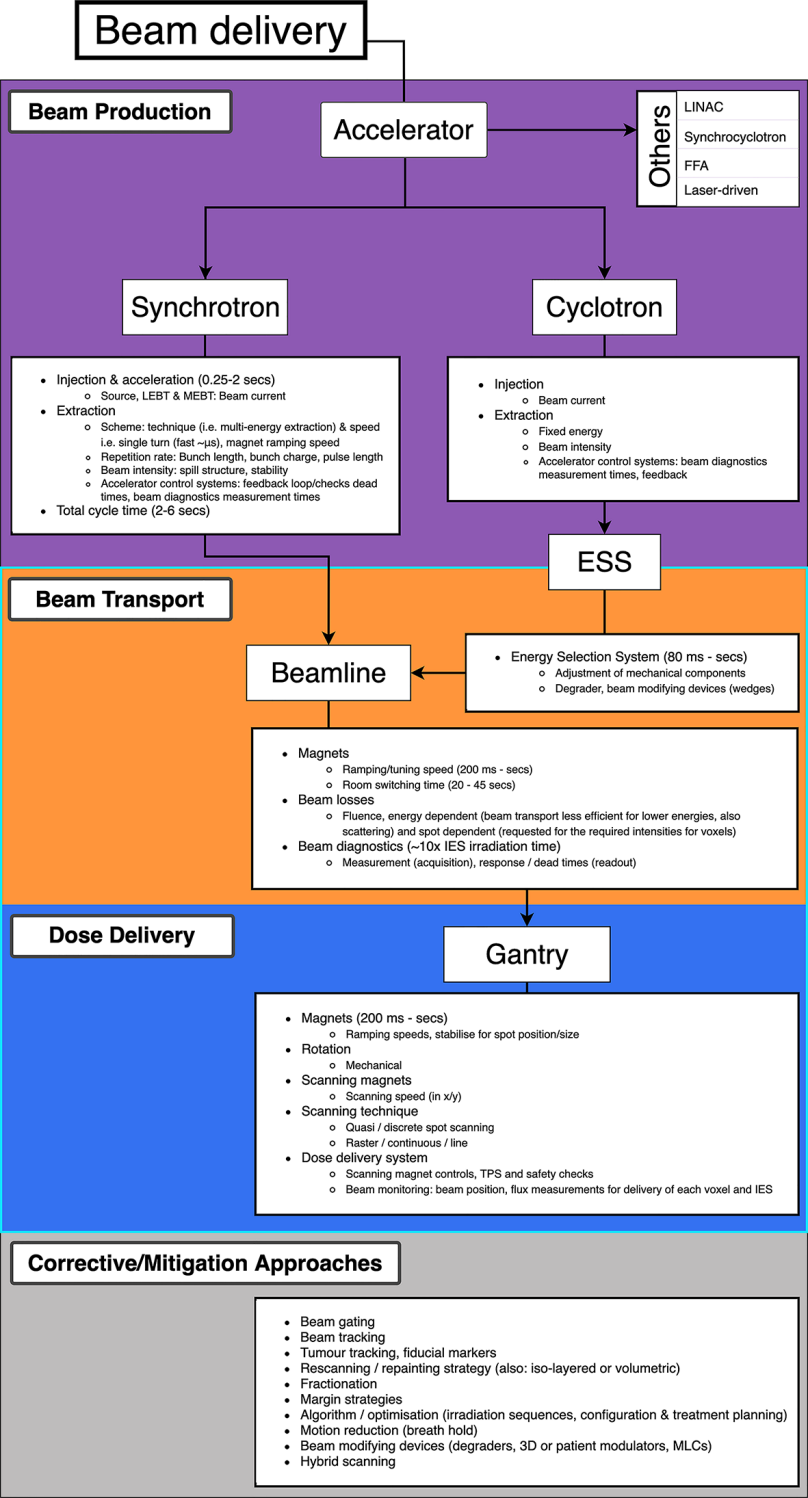
Overview of PBS beam delivery and different motion mitigation strategies. Breakdown of contributions to the BDT from the beam production, beam transport and dose delivery processes.

Decreasing BDT is complex as prescribed treatment plans are not standardized: the BDS, accelerator and other systems vary at each facility and the delivery efficiency depends on numerous technology-related factors. Facilities have different equipment vendors, number of rooms, delivery systems etc. and often adopt different processes: these characteristics all influence the delivery procedures implemented (27). Meanwhile the treatment plan calculates the number of spots and layers to deliver the required dose distribution to the target volume. Nystrom & Paganetti et al. (13) state the three main components which constitute the delivery time for a treatment field: time to irradiate a spot, time to move between spots and the time to change beam energy. Speeding up any of these components can shorten BDT however they are not independent variables. Delivering a faster treatment is not straightforward; it is not solely dependent on the capabilities of the BDS itself but is a multi-faceted problem. The contributing factors which impact the dose delivery and BDT are illustrated in **Figure 2**.

### 2.1 Pencil Beam Scanning

**Figure 3:**
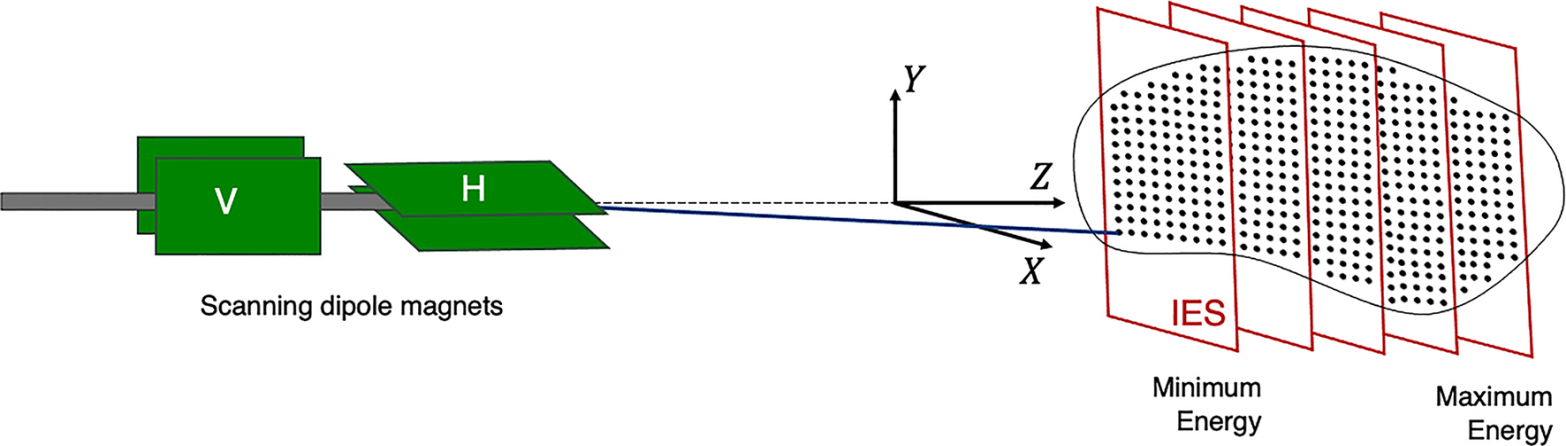
Active PBS. The BDS delivers a conformal dose distribution to the treatment volume by scanning the beam along a calculated path in the transverse plane. The beam energy is then adjusted to change the depth (typically lower in energy), switching to a proximal IES, scanning through subsequent layers.

The planned dose distribution determines the requested parameters within the limits of the source, accelerator and beam transport lines, including the beam energies, size at isocentre, intensities and delivery channels. Each different configuration can total to thousands of available beam combinations (28), these multiple beams produce a 3D dose distribution where the entire process can amount to long treatment times. For PBS delivery, the beam is magnetically deflected across the tumor in the transverse plane across one layer or an iso-energy slice (IES), then adjusted longitudinally to a shorter depth (typically a decrease in proton range of 5 mm in water) and repeated (**Figure 3**).

**Figure 4:**
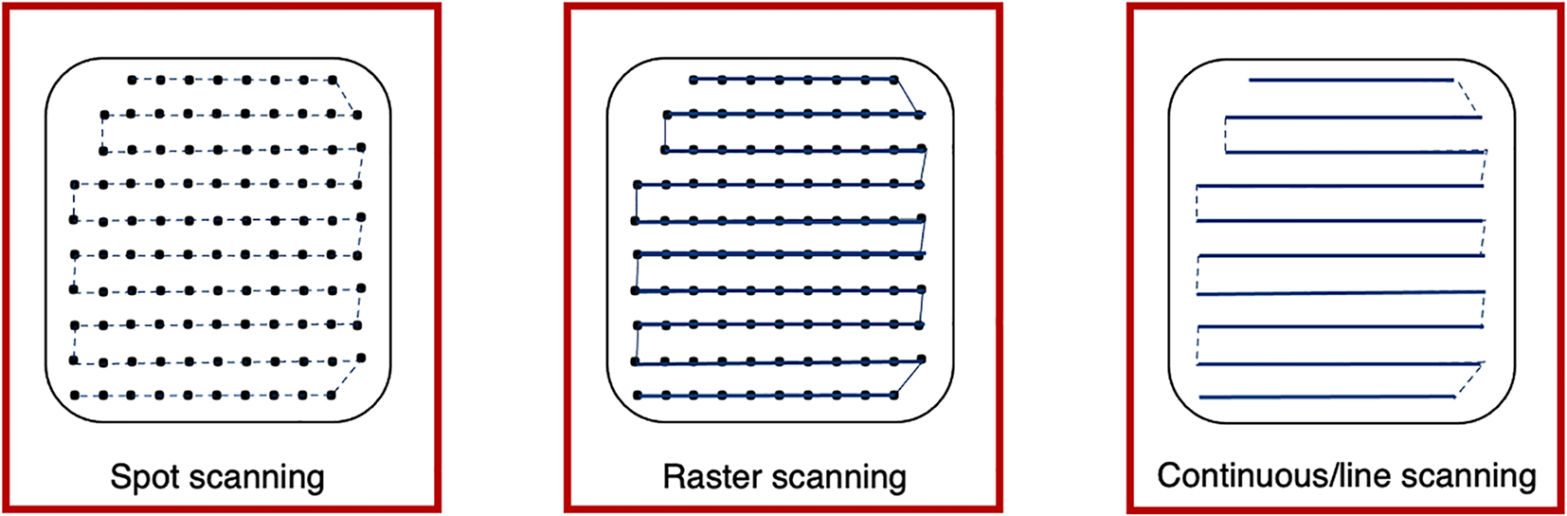
Spot, raster and continuous line scanning patterns for PBS delivery. Spots and solid lines indicate beam-on irradiation and dashed lines indicate movement with beam-off. For spot scanning, the beam is turned off between movement to the next spot but remains on for the whole delivery in raster scanning. For continuous line scanning, the dose is delivered across a linear path (rather than as spots) and may be turned off between movement to subsequent lines.

Different scanning techniques (spot, raster and line/continuous) may be used with optimization methods to deliver the beam and irradiate each layer. Spot and raster scanning is usually dose driven, where progression through the spots occur when the prescribed number of protons have been delivered. Continuous line scanning moves the beam according to a calculated path dependent on time and consequently, may be affected by beam current variations (29). This requires a continuous beam, where the quick delivery also makes it more challenging to validate and monitor (13). For all techniques, the dose is painted such that the accumulation of the distribution in both planes results in sufficient tumor volume coverage (**Figure 4**).

### 2.2 BDT Time Components

**Figure 5:**
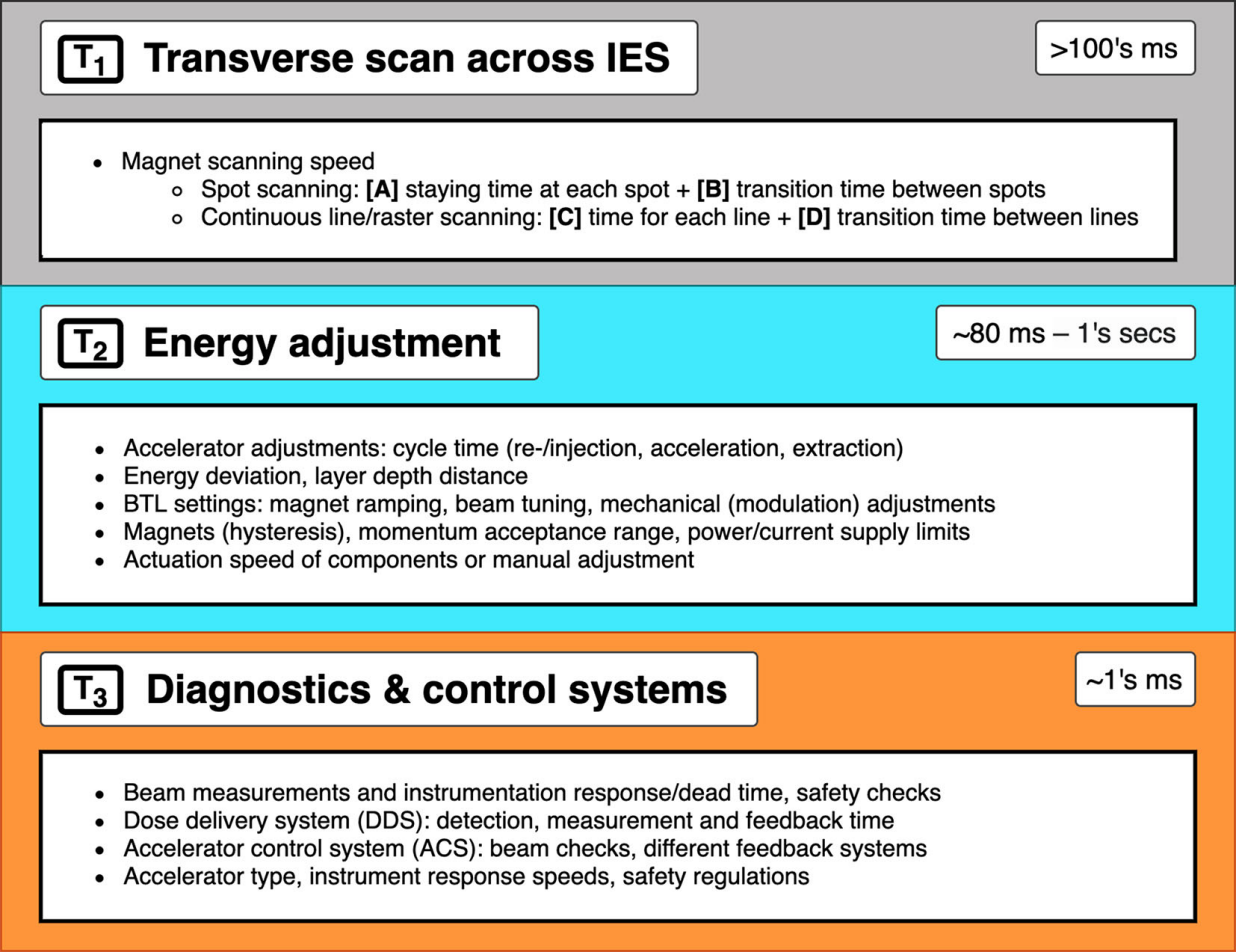
Major BDS components and corresponding factors which contribute to BDT.

The delivery of a field combines multiple aspects of the BDS: if we consider only the beam delivery process, this can be approximated to include the **[T_1_]** transverse scanning, **[T_2_]** energy adjustment and **[T_3_]** systematic dead times (**Figure 5**). These estimations are provided (within applicable orders of magnitude) as based on broadly reported values.

The **[T_1A_]** staying or ‘*dwell*’ time at a position varies according to the intended amount of dose (and extracted beam intensity); it can be down to 0.1 ms (30) however is likely to be of the order of 1’s ms (31). The **[T_1B_]** transition time to the next position can be ~3 ms (29, 32) or ~10 ms between raster points (33). For line scanning, **[T_1C_]** could be 5 ms for a line and **[T_1D_]** 5 ms to move to the next line (34). Irradiation time for a single IES **[T_1_]** depends on the size of the distribution: it increases linearly with the number of spots, likely needing at least ~100 ms.

**[T_2_]** ranges from 80 ms to a few seconds (16), faster energy modulation is possible with cyclotrons and synchrocyclotrons, and slower direct energy adjustments with synchrotrons. This is discussed in detail later in Section 3.2; the fastest reported ELSTs are listed in **Table 1**.

**Table 1:** Reported minimum ELSTs ([T2]) for currently used clinical cyclotron, slow cycling synchrotron and synchrocyclotron accelerators.

	Accelerator type		
	Cyclotron	Synchrotron	Synchrocyclotron
Energy layer switching times – fastest reported Modulation method	PSI G2: 80 ms G3: 200 ms ESS, degrader (carbon wedges)	HIMAC MEE: 220 ms Hybrid: 100 ms MEE (>3 cm depths) and range shifters (<3 cm)	Mevion S250i 50 ms Motorised modulator plates (lexan/polycarbonate), 2.1 mm depth change
References	(35, 36)	(37–39)	(40)

**[T_3_]** ionization chamber measurement times average ~0.1 ms (13, 28). ACS and diagnostic safety checks ensure correct beam parameters (spot size, position, intensity etc.). As reported by Schoemers et al. (41), measurement times are a bottom line limit across the BDS and similarly for continuous scanning, the lower bound is determined by the instrumentation speed (21); at least 1 ms for an IES is required.

The BDT is a function of the irradiation sequencing and indeed, a larger tumor volume or higher number of IES recruits more of these actions **[T_1–3_]**, amounting to a longer BDT. Independently, the irradiation time is also determined by the intensity of the beam produced by the accelerator (16, 41). As a standard (PBT) clinical minimum, most facilities have the capability of delivering dose rates of 2 Gy/min to a 1 L volume, 10–20 cm deep (42). This equates to beam currents of 100’s nA at the patient, varying for accelerator type. However, even at facilities which are able to achieve higher dose rates, there are practical limitations with operation at higher intensities (Section 3.1).

## 3 Limitations and Avenues For Improvements

### 3.1 Accelerators

**Figure 6:**
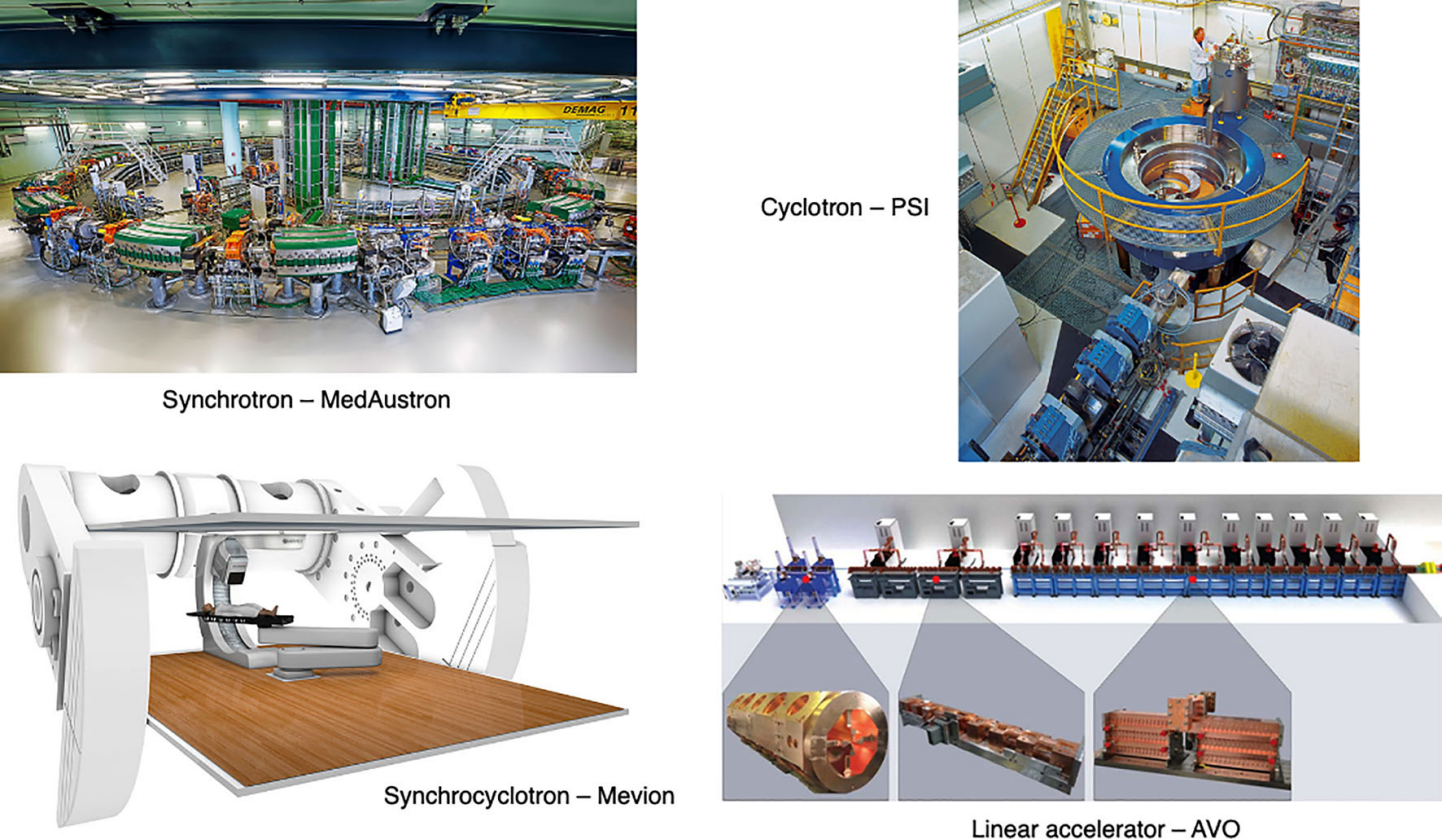
Synchrotron at the MedAustron facility (43) and COMET cyclotron (for PBT) at PSI (44). Gantry-mounted Mevion S250i synchrocyclotron (Mevion Medical Systems, Inc.). Depiction of the AVO LIGHT LINAC with the radiofrequency quadrupole, side coupled drift tube LINAC and coupled cavity LINAC sections highlighted (45).

The timing structure of delivered beams varies significantly between different types of accelerators, as they have different technological and safety limitations. In this section we present the main operational patterns and optimization challenges for synchrotrons, cyclotrons, synchrocyclotrons and other accelerators that may soon be available for PBT or CPT facilities (**Figure 6**).

#### 3.1.1 Synchrotrons

The most common operation pattern for synchrotrons starts by injecting a beam pulse from a radiofrequency quadrupole (RFQ) and linear accelerator (LINAC) at up to ~5 MeV/u (order of 10–100 μs) of ~10^11^ protons (or ~10^9^ carbon ions) into the main synchrotron ring. The beam is accelerated in ~1 s to the desired energy, then slowly but continuously extracted and transported to the irradiation room to treat a specific treatment slice. While extraction processes are typically capable to deliver the entire accumulated charge in <100 ms, the process is intentionally extended to safely match the maximum supported scanning speeds in the transverse plane.

After delivery to one IES is completed, the particles still circulating in the synchrotron are dumped and all the magnets of the main ring are ramped to their maximum current and back to injection levels in typically ~1–2 s. This process, often called ‘*magnet washing*’ (~100’s ms), ensures reproducibility of the magnetic field in the presence of hysteresis of ferromagnetic components. If the number of particles injected into the synchrotron is not sufficient to deliver the required dose at the specific energy, more pulses will be injected, accelerated, and delivered. For injection pulses of the order of ~10^11^ protons, only the treatment of large lesions (>1 L) in combination with hypofractionation requires more than one injection for the IES. The total time required to change energy (or refill the main ring) is of the order of ~2–4 s.

Two significant technological solutions exist which can drastically reduce the time required to change energy or refill the ring. This can be shortened to ~1–2 s by decreasing the time required to wash the magnets by employing an active regulation of the magnet power supply output, based on live measurement of the magnetic field (46, 47). However the technology necessary for active regulation of quadrupoles or sextupoles has not yet been implemented in clinical machines.

Another technique is called multiple energy extraction (MEE) operation (or extended flattop operation) and aims to reducing only (but drastically) the time required to change energy. In MEE (48) the beam can be extracted across several energy levels in a single spill; this enables the delivery of successive IES without needing to wait for re-/acceleration. The unused part of the beam circulating after completion of a slice is re-accelerated (or decelerated) instead of being dumped in preparation for a re-injection. Although the process is not completely lossless, it requires only roughly ~100 ms (37, 49) to change beam energy^2^. Younkin et al. (50) performed a study to quantify BDT savings with MEE implemented at a synchrotron PBT facility, finding an average BDT reduction of 35%. The ELST was reduced by ~90% from ~2 s to 200 ms with MEE. Additional savings could also be achieved by improving charge, extraction limits and charge recapture rates: these depend on the performance and limits of the synchrotron^3^.

The extraction mechanism most often used in synchrotron ion beam therapy facilities is based around two methods: a slow resonant mechanism which is usually driven by a transverse excitation [RF knockout (RF-KO) (51, 52)] or a longitudinally slowly induced energy change [Betatron magnet (47, 53, 54)]. For slow resonant extraction, the beam can be kept bunched throughout the extraction process, making re-acceleration (or deceleration) a delicate but feasible process. However, this produces fundamentally different beam distributions in the horizontal and vertical plane. This requires an optics matching stage in the BTL, often realized by inserting a thin foil that spreads the beam to equalize the particle distributions. The foil orientation might also require mechanical adjustment at different energies, potentially limiting the ELST^4^.

The second technique requires the de-bunching of the beam, which makes further re-acceleration (or deceleration) theoretically possible (RF front acceleration), but extremely challenging, loss prone and potentially time consuming. Facilities not originally designed for MEE operation exhibit the trend to implement RF-KO first rather than attempting other retro-fitted techniques (56–58). The direction of energy change is typically fixed (e.g. always increases or decreases), because an up-down energy scanning would violate the reproducibility of the main ring magnetic field due to hysteresis effects. A magnetic field active regulation control would be necessary for this feature.

Alternative extraction techniques which are compatible with bunched beams are based on optics changes often used in larger synchrotrons for non-clinical applications (59). These could be applied in the future but so far promise limited advantages for small machines dedicated to therapy. The limited benefits offered by MEE operations for hypofractionated treatments can be overcome by increasing the particle filling of the ring.

It is not uncommon for an IES to host a vast dynamic range of dose among its spots. If the beam intensity is kept constant, it has to be as low as necessary to fulfil a minimum dwell time on the lowest rated spot. Synchrotrons that adopt RF-KO can regulate the extraction speed of the accumulated charge spot-by-spot, with fast feedforward loops (60, 61). The properties of the beam are largely independent of the strength of the field applied. This feature can reduce the BDT considerably and is already implemented in most of the latest generation commercial solutions.

Although it is not the focus of this work, it is worth mentioning that synchrotrons are often chosen by therapy centres also for their capability and ease of delivering multiple particle species (proton, He, C, O, etc.), assuming multiple ion sources are used for each species. The time required to switch particle species is driven by the change and stabilization of the field in the injector and low/medium energy BTL magnets. In some tests at NIRS-QST this was chosen to be ~20 s (62) but could potentially be reduced to only a few seconds with a dedicated design and development of the source, injector and overall control system (63).

#### 3.1.2 Cyclotrons & Synchrocyclotrons

The most common choice for PBT is the cyclotron (mostly isochronous cyclotrons), which accelerates protons to a single (maximum) energy^5^. The beam is typically available as a continuous wave beam with a micro-bunch structure of 100’s MHz. As the extraction energy is fixed, material must be inserted in the beamline to change the energy. This produces large losses [i.e. >99% of the beam can be lost (66)] especially when selecting lower energies, resulting in a radioactive hot spot and a beam with a very large distribution of energies, not suitable for precise 3D conformal dose delivery. Therefore, an ESS usually follows the energy degrader, consisting of several devices (degraders i.e. carbon or graphite wedges, collimators, slits, magnets, diagnostics etc.) which are necessary to modify the beam for the correct parameters for treatment. The transmission, quality and distribution of the beam is affected by interactions with objects in the beam path (the increase in distal penumbra from the energy spread can be up to 10 mm) (16). The optics is designed to create a section with a large dispersion, where slits are inserted to trim the beam and reduce the energy spread.

Compared to synchrotrons, the time taken to mechanically insert the beam modifying devices is relatively quick (~10’s ms) for small energy adjustments. The use of actuated static wedges with time compensation and fast deflecting magnets (range adaptation) is reported to be the fastest method, changing energies in less than 20 ms (67). At PSI the wedge positioning takes 50 ms; Pedroni et al. (35) report the fastest energy modulation times are achievable on gantry 2 (G2) at 80 ms. Delays are caused by stabilization of the dipole magnets in the BTL and gantry. The direction of the energy change is not limited by the accelerator (can change up and down in any sequence), but the reproducibility of the magnetic field in the ESS. Thehysteresis of ferromagnetic components in the BTL typically restricts fast energy change to one direction only, with a magnet wash required before each direction swap. The BDS magnets must be ramped to accommodate different energies: the time taken to vary and reset the magnetic fields in the BDS determine the ELST time. This also holds true for synchrotron facilities however, the times required for energy changes with the accelerator far surpass these at present.

Spot-by-spot intensity modulation (as done with synchrotrons) is possible, but more complex to achieve for cyclotrons and LINACs because they do not accumulate charge. The dose rate can be regulated by either modifying the extracted current from the ion source or by forcefully reducing the transmission throughout the accelerator. Although many parameters can be found in every design that could achieve this goal, it is extremely difficult to avoid affecting other beam properties at the same time (68, 69), especially in the very wide dynamic range (2 orders of magnitude) required to fully exploit this feature.

Superconducting synchrocyclotrons produce a pulsed beam (few μs, every 1–2 ms) as they are not isochronous (42, 70). Only one or a few pulses-per-spot is usually necessary. Synchrocyclotrons can bypass the limiting constraints of the BDS magnets with single room systems (for PBT) where the accelerator is gantry-mounted and the entire machine rotates around the patient. Energy changes are performed using an energy modulation system; like a regular cyclotron this comprises polycarbonate plates, range shifters, absorbers or other devices which physically attenuate the beam (71, 72). ELSTs as fast as ~50 ms, for changes of 2.1 mm in water equivalent thickness have been achieved (40). Although synchrocyclotrons have a smaller footprint and fast energy modulation, the achievable beam parameters and pulse structure are insufficient for continuous PBS delivery (13).

#### 3.1.3 LINACs, Laser-Driven & Fixed Field Alternating Gradient Accelerators

Linear accelerators are already ubiquitous in hospitals as compact sources for conventional XRT. Using a LINAC for protons or ions is more challenging: they are physically much larger, in part because the velocity of the particles changes significantly in the clinical energy range, thus the physical length of accelerating gaps must accommodate for this throughout the accelerator. A proposed LINAC-based solution from Advanced Oncotherapy (73) includes: a high-frequency RF quadrupole design at 750 MHz, originating from CERN, and a side-coupled 3 GHz LINAC for the high-energy accelerating section originating from the TERA foundation (74, 75). Above a threshold minimum energy (~70 MeV for protons),modular cavities are used to enable the LINAC to change energy. This is an unusual LINAC design, but enables the beam energy to be precisely regulated pulse-by-pulse at rates of ~200 Hz. This translates into a minimum time for energy change of just 5 ms (16). Technology capable of supporting kHz pulse rates for lower ELSTs exists. A LINAC is capable of switching energy in any direction but the remaining bottleneck would be the magnetic reproducibility in the BDS.

Recent studies on clinical suitability for LINACs show that the production of a stable spot size with energy can result in increased conformity particularly for deep tumors (76), and the ability to vary spot size on demand while delivering protons at FLASH dose rates could lead to LINACs having greater conformity and larger tumor volume capability compared to cyclotrons (77).

Additionally, LINAC designs could also be adjusted to accelerate different particle species, where particle switching times are limited by magnetic field changes and stabilisation in the ion source and low energy section. Cavities with increasingly higher accelerating gradients are being designed and proposed, exploiting synergies with accelerators developed for high energy particle physics experiments. This trend could also contribute to shrinking the injector stage of synchrotrons. Although the most pursued R&D is a reduction of the facility footprint, LINACs capable of accelerating orders of magnitude larger currents of protons and light ions to ~10 MeV exist or are being developed^6^ (79–81).

Another concept which has been considered are laser-driven accelerators. Although much interest has been generated since the early 2000s, progress has been slower than initially anticipated, primarily due to limitations in laser repetition rate, beam intensity, control and reproducibility (82). Proposals exist to use laser-driven ions both as a pre-accelerator into a synchrotron (83) – in which case beam delivery aspects remain as they are at present – or more radically to replace the entire magnetic beam delivery system with an optical one (84). Compared to conventional ion sources, laser-driven beams have large energy spread or even an exponential energy distribution over the full energy range and large divergence, which makes efficient beam transport very challenging (85). A realistic implementation would likely require a beam capture, ESS, collimation and BDS. Existing studies focusing on protons only have proposed high field (>8 T) pulsed solenoids for beam capture, although in some cases a conventional quadrupole triplet could be considered (86, 87). This latter option faces challenges in controlling ion species, energy spread, energy control and other aspects (88).

Alternatively, a potential future option is the Fixed Field Alternating Gradient (FFA) accelerator, which has static (in time) magnets but a beam orbit which spirals slightly outward with increasing beam energy^7^. Unlike the cyclotron, the energy range of the FFA is in principle limitless, so heavier ions including carbon can also be accelerated to clinical energies.

FFAs have many potential applications as they can be compact in size, with fast acceleration and high beam current (89). The largest limitation is that this technology is less well-established than synchrotrons or cyclotrons: few accelerator physicists, engineers and component suppliers are familiar with this type of machine. However, they were first proposed back in the 1960s and have been developing rapidly in the last two decades. A number of FFAs have been constructed, the most relevant being the two 150 MeV proton FFAs constructed in the early 2000s in Japan (90) with 100 Hz repetition rates which have been the subject of detailed beam studies and characterisation (91). New designs for proton and ion therapy, including superconducting designs, have not yet been prototyped or constructed.

In general this type of accelerator produces a pulsed beam. This leads to a key advantage of the FFA for charged particle therapy: fast variable energy extraction, usually with single-turn extraction^8^. Extraction can occur at any time in the acceleration process, dictating the beam energy. A second advantage is that the FFA removes magnet ramping, overcoming hysteresis or magnet washing issues, so the pulse repetition rate of the FFA can be much higher than a synchrotron: a rate of 1 kHz was the goal of a 2010 design study PAMELA (Particle Accelerator for MEdicaL Applications) (92). This is vastly different to the few seconds (<1 Hz) for a regular synchrotron or 50–70 Hz for a rapid cycling synchrotron. This rapid pulse rate has a remarkable feature of enabling pulse-by-pulse flexibility across the entire clinical energy range without limitation in energy step or direction, with a choice of particle species. This could reduce the ELST to the order of ms and also enable the possibility of interleaved treatment pulses (carbon or proton) and imaging (high energy proton) pulses delivered through the same BDS.

Removing restrictions in energy variation imposed by existing technologies could open up treatment options that are impossible at present. Nonetheless, taking full advantage of the rapid energy changes enabled by FFAs, LINACs or other machines with fast energy variation would require the BTL and/or gantry to be able to accept and deliver the extracted beam.

### 3.2 Energy Layer Switching Time

The transverse motion of the beam can be sped up by using continuous scanning methods and with faster dipole magnets. As discussed by Flanz & Paganetti et al. (13) scanning dipoles from 3–100 Hz are used clinically but capabilities also depend on their size, distance from the patient as well as inductance and power supply considerations. Additionally, speeds are restricted by the viability of currently available beam instrumentation tools to accurately and rapidly, measure and record dose rates.

While the transverse scanning magnets are relatively fast; it can take ~100 times longer to move the beam the same distance longitudinally. Decreasing the time to change energies between IES is a challenging issue. It is not just a singular aspect of the delivery process but is governed by several factors: primarily the accelerator and the BDS. The ELST ranges widely across facilities and can be up to an order of magnitude longer than the time it takes to scan across an IES. A comparison of minimum baseline figures for clinical accelerators are shown in **Table 1**.

If we consider the breakdown of time components again (Section 2.2), the BDT can be approximated by summing the different contributions. Assuming some general conditions (uniform, continuous beam intensity) with a typical PBS **[T_1A+B_]** spot delivery speed of 6.67 ms/spot [i.e. 150 spots/s; PSI report 5 ms/spot = 200 spots/s (93)]. As a practical example, we take values from a robust IMPT “standard clinical plan” for an oropharyngeal case, averaged for 5 patients over 3 fields (94) where 700 spots are needed over 42 layers. Therefore, a BDT estimate^9^ with a typical ELST (1 s), for a single field irradiation: 42×[**T_1_** = 113 ms] + 41×[**T_2_** = 1 s] + 42×[**T_3_** = 2 ms] = 45.84 s.

**Figure 7:**
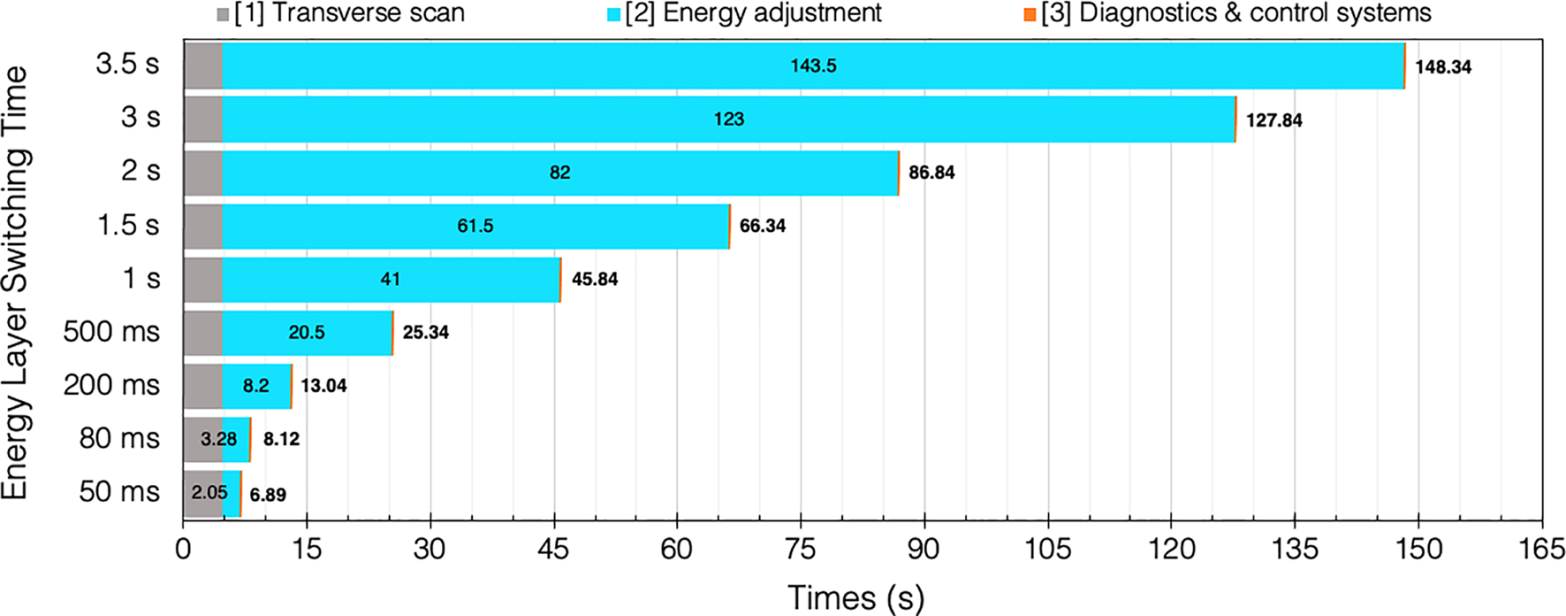
BDT estimates and timing contributions for an example head and neck case (94) given a range of clinical ELSTs [T2]. Transvers scan [T1 = 4.76 s] and system dead times [T3 = 0.08 s] are assumed constant for each of the 42 IES.

For comparison, if a fast ELST (80 ms) is instead applied: 42×[**T_1_** = 113 ms] + 41×[**T_2_** = 80 ms] + 42×[**T_3_** = 2 ms] = 8.12 s. Evidently, this results in a much shorter BDT; particularly for complex cases with many layers which may also need rescanning, there is an accumulation of time saved for each IES. The increasing penalty for longer ELSTs on BDT for this case is illustrated in **Figure 7**.

Several studies have been performed which examine the time components quantitatively and evaluate the impact of the ELST on BDT, as a means to improve treatment delivery efficiency. Shen et al. (22) carried out a detailed analysis to model the BDT at a synchrotron PBT facility based on operational parameters including the ELST, average scanning speeds, spill rate, charge and extraction time, magnet preparation and verification time. The average ELST across the energy range and scanning speeds in *x* and *y* were reported as 1.91 s, 5.9 m/s and 19.3 m/s, respectively. Values determined by the model were compared with log files from a large range of delivered patient treatments to calculate the contributions to BDT. The ELST was identified as the most dominant contributor to BDT at 71%; reducing this time would greatly improve beam utility during delivery.

All components of the PBT treatment process were also comprehensively analyzed by Suzuki et al. (26) to evaluate the use factor and efficiency of beam delivery parameters for different disease sites. Although there are numerous factors, the BDS largely governs treatment efficiency which is asserted as the most important factor as it is directly related to utility and availability. For facilities which operate a busy schedule, reducing BDT can enable greater throughput; at this clinic, a 1 min reduction in BDT for a single field accumulated to treating an equivalent 10 more prostate patients a day. This can also lower costs as treatment costs scale with the total time spent by the patient in the treatment room (13).

Increasing throughput is an important consideration to improve the availability of CPT. A sensitivity analysis of daily throughput capacity – and therefore efficiency of PBS treatments – was subsequently performed at the same facility by Suzuki et al. (23). Several parameters in the treatment process were similarly studied; the BDT was reduced to the sum of the ELST and spot delivery time as a function of the treatment volume, dependent on the disease site. The ELST was reported as 2.1 s and accounted for 70–90% of the BDT for the majority of tumor volumes (<1 L). Although for this case the BDT is limited by the accelerator, a reduction in the waiting times can greatly decrease BDT: the ELST as well as room switching time account fora large part of the total treatment time. Increasing the uptime by minimizing beam-off time can significantly improve throughput (95). Nystrom and Paganetti et al. (13) emphasize that improvements in this area will have the greatest efficiency gain and that a shorter BDT will have the greatest impact on facilities with multiple rooms.

An optimization which could further reduce BDT is by splitting the beam for delivery to multiple different rooms simultaneously. This is more realistically achievable with accelerators carrying a microbunched beam structure. In this case transverse RF fields could be used to induce initial beam separation in transport lines. However, high beam intensities and continuous, reliable operation require additional degraders and shielding. The complexity and cost of the facility, ACS and other systems would nonetheless increase considerably and safety must also comply with medical standards (13, 96). Developments in the BDS and accelerator technology are needed for this; a more practical pursuit could be to optimize processes surrounding treatment set-up, room scheduling and utilization.

### 3.3 Beam Delivery Systems, Gantries & Fixed Beamlines

At clinical facilities, the ELST typically approaches the order of seconds with a commercially available BDS, much longer than the baseline values reported in **Table 1**. The time delays to change the magnetic parameters of the beamline are a bottleneck for cyclotron facilities; for synchrotrons, the use of MEE has been implemented in specific instances but is not yet universal (50). Nonetheless, it is clear that the ELST is contingent on magnet ramping speeds and will be prohibitive, particularly when considering emerging developments in the field. In fact, the cost of accelerators is in general lower than the BDS so improvements in accelerators alone will not be sufficient for CPT to reach levels of XRT adoption (25). Accordingly, Myers et al. (18) have advocated that progress with accelerators need to be matched by the BDS in order to accommodate for fast energy variation. Two possibilities are suggested: the use of superior magnets or alternatively, to increase the energy acceptance range. The first option would involve low inductance magnets that require large currents and therefore higher build and running costs. The second option has been considered frequently in literature (96–98) and is becoming more of a possibility with the realization of superconducting (SC) technology and advanced magnet designs.

**Figure 8:**
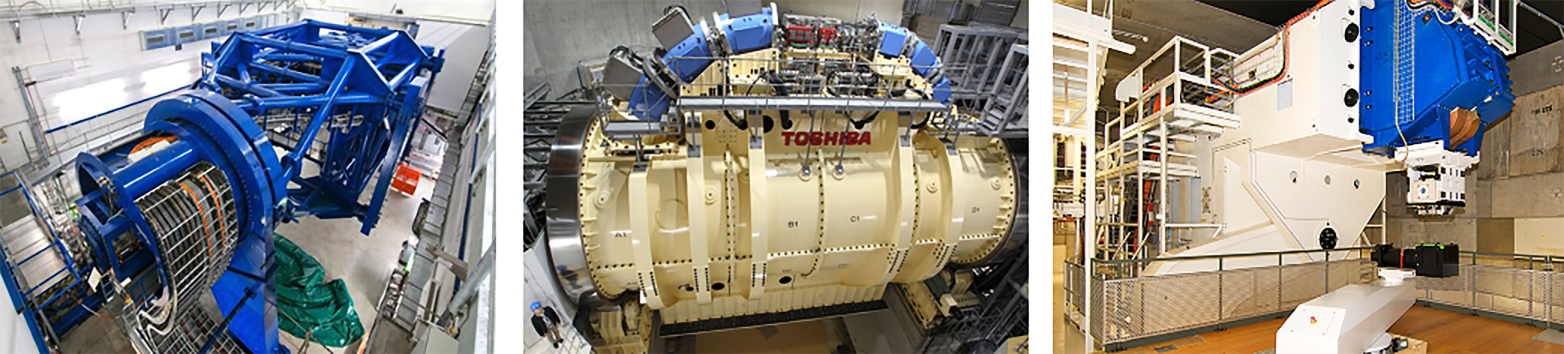
Gantry installations at HIT (106) and HIMAC, QST (104) for CIBT. PSI PBT Gantry 2 (Photo: Paul Scherrer Institute/Markus Fischer).

The BDS contributes to a significant share of the capital and total cost for a CPT facility (24, 25) so future iterations must be designed to reduce operational and construction costs. The BDS must be able to transport the beam with high accuracy (sub-mm precision), at different specific energies and deliver the correct dose distribution reproducibly. For systems which possess a gantry, the downstream section of the BDS comprises of series of magnets to bend and transport the beam to isocentre with the required treatment parameters. The entire gantry rotates to deliver the beam from multiple entry angles. Consequently, the gantry (**Figure 8**) is a physically large and complex mechanical structure: this amounts to considerable costs associated with the weight, size, construction and operation. Most modern proton facilities have gantries in order to deliver PBS which achieves the highest quality of treatment.

For heavier ions, costs are much higher as the gantry must accommodate particles with larger beam rigidities and added physical constraints introduce greater probability of errors (99). Currently there are only a few facilities which deliver carbon-ion beam therapy (CIBT) using a gantry: HIT, Heidelberg, Germany has a gantry which has a footprint of 6.5 m × 25 m (radius × length), weighing ~670 t (100–102) and HIMAC, QST, Japan has a SC gantry, 5.5 m × 13 m weighing ~300 t (103, 104). A second generation, compact SC gantry with a smaller 4 m × 5.1 m footprint was also developed with Toshiba (105). Heavy ion facilities which do not employ a gantry are limited to delivery with fixed beamlines.

The use of SC magnets can dramatically decrease the weight and size of the gantry as higher fields (necessary for >1.8 T) can be achieved with comparatively fewer and smaller magnets. However, the costs for the magnets themselves and the operation of cooling systems may not be economical (98).

These further challenges and costs associated with delivering heavier ions with a gantry hinder its practicality. As such, the question of the necessity of a gantry itself has now been raised: Flanz & Paganetti et al. (13) propose that the simplest way to reduce costs is to remove the gantry completely and in place have a fixed beamline. A PBT study by Yan et al. (107) indicates that for several disease sites (PBS head and neck cases), treatments could effectively be delivered gantry-less, requiring only a few fields with fixed geometries. There are other potential benefits to removing the gantry besides lower costs (maintenance, commissioning and also construction i.e. shielding) and the use of upright chairs is now being reconsidered. Seated positioning is typical for ocular treatments and for some specific disease sites; it was also historically the method used at pioneering facilities yet with less success than supine treatments (108). Now, with the advent of modern delivery techniques, superior dose distributions can be achieved with seated treatments and clinical advantages with better immobilisation have also been reported (109). The use of vertical CT enables imaging of patients with the same treatment position for treatment planning and positioning errors can also be corrected with geometrical adjustments of the chair (i.e. changing pitch) (14). This could be particularly effective for tumor sites which are difficult to treat due to motion and could also provide better patient comfort.

A study by Sheng et al. (110) report that rotational and translational positioning with a 6D treatment chair is comparable in alignment precision and reproducibility to a standard robotic treatment couch. Clinical tests of this chair were performed by Sun et al. (111) to verify treatment and workflow feasibility. As optimal plans are possible with only a few fields, it is suggested that beam selection is more significant to the achievable dose distribution than the available number of fields or angles. Additional imaging is also required to ensure correct patient positioning however results showed similar intrafractional deviation to treatments in a lying position. The increased physical demand of a seated treatment indicated the need for better immobilisation procedures; an interesting prospect is if motion or positioning differences could be further reduced. Nevertheless, overall patient comfort is an important consideration for upright treatments and Mazal et al. (109) list several cases where there could be advantages, such as increased ease with patient anesthesia or airway management. Furthermore, the increased availability of physical space with a fixed beamline also enables greater flexibility with the beam optics and delivery components. This could allow the BDS to produce a beam with a wider range of characteristics (field size, spot size, scanning capabilities etc.) as the optical design and inclusion of auxiliary devices can be reconsidered without the conditions imposed by the rotational and mechanical constraints of the gantry. Similarly, another significant benefit is the prospect of enhanced integration with imaging; improved conformity and registration between imaging modalities and the possibility of online imaging systems. This technology is currently being commercially developed and the option of upright treatments with a gantry-less system in clinical practice may soon be a possibility.

#### 3.3.1 Energy Acceptance Range

The energy acceptance – or momentum acceptance – is a limiting factor in existing beamlines and gantries. A typical momentum acceptance range is ±1% (approximately ±2% energy acceptance), equating to changes of 5 mm in water equivalent depth; this is the usual spacing between each adjacent IES. This acceptance band is a technical limit corresponding to the maximum deviation from nominal beam momentum which can stably be transported by the optics. Any such momentum deviation produces a change in trajectory (via dispersion) and the configuration of the magnetic elements determines the particle beam dynamics and stable range.

Presently, the settings of all the BDS magnets must be changed synchronously for each IES, whilst considering AC losses and hysteresis effects, requiring several checks and settling time for field stability (98). This preserves the correct beam parameters at isocentre and ramping typically occurs in one direction to reduce complexities. SC magnets with high ramp rates also experience issues with eddy currents, but their use in the BDS for heavier ions appears necessary in order to minimize size and weight. Increasing the momentum acceptance range enables the BDS to transport various beams with the same fixed magnet settings and therefore minimal dependence on their field ramping capabilities.

Several designs for PBT have been proposed which use achromatic beam optics to suppress dispersion effects, reporting momentum acceptance ranges of ±3% by Gerbershagen et al. (112), ±15% by Nesteruk et al. (113) and ±25% by Wan et al. (114). For heavy ions, large acceptance can be achieved with new SC magnet designs (canted-cosine-theta combined function magnets) (14).

**Figure 9:**
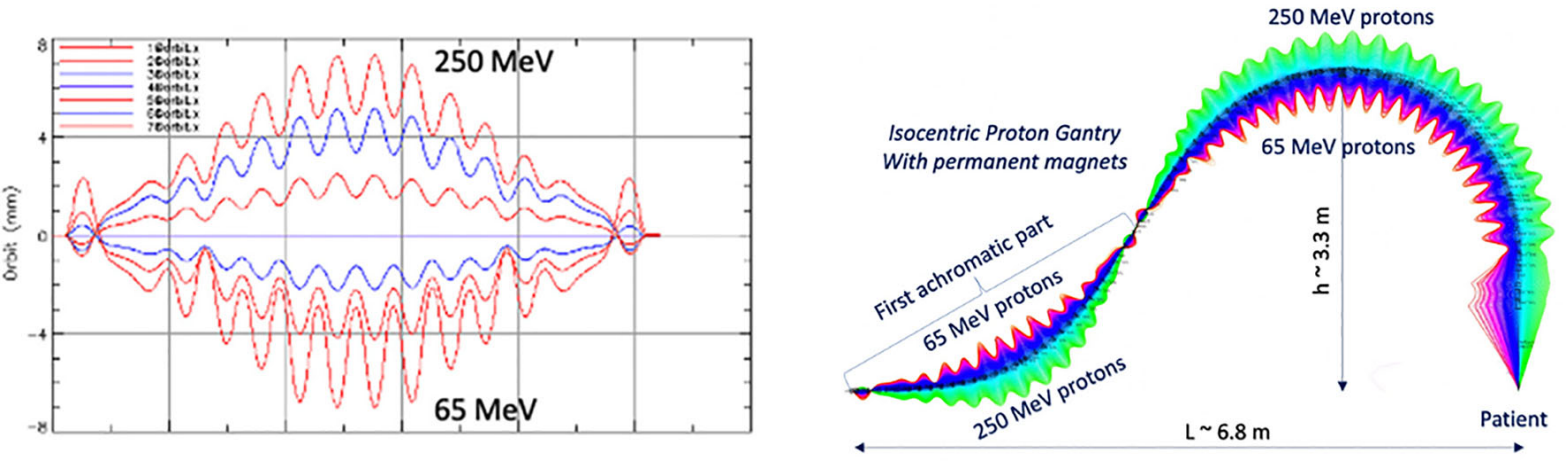
Orbit shape with varying energy (left) showing an energy acceptance range of 65–250 MeV. Orbit offsets within the permanent FFA gantry for PBT (right). Note the orbit offsets with energy are magnified for clarity in the right hand image and are around 15 mm, as shown on the left (117).

Another optical configuration which enables a large energy acceptance (LEA) is the FFA concept (Section 3.1.3). With non-scaling FFA optics, combined function dipole and quadrupole magnets can be arranged in repeated cells in an alternating gradient configuration, resulting in strong focusing in both planes with small dispersion. This is stable for a wide range of energies and enables beam traversal along the beamline at multiple physical positions within the same fixed magnetic fields. Due to the low dispersion, small aperture magnets can be constructed, minimizing size and construction costs. Multiple designs using FFA optics have been reported by Trbojevic et al. (115, 116) with a momentum acceptance range of approximately ±20–30% using SC magnets for both PBT and CIBT. Alternatively, novel Halbach type permanent magnets have been designed for a PBT gantry (**Figure 9**) with a footprint of ~2.2 m × 7m, accepting up to ±35% (117, 118).

Alternative gantry designs to allow rapid beam delivery also include a novel method using high field SC magnets to produce a toroidal field capable of delivering beams from multiple directions in a fixed, steady state, ‘*GaToroid*’ gantry (119). This removes the mechanical and time constraints typically required to change angle and energy. However, there may be limitations with the number of delivery angles, field size and challenges in achieving positional accuracy. Furthermore, several aspects with the design, engineering, field configuration, beam transport and optical modelling are still under development.

In general, increasing the energy acceptance of a BDS to enable a LEA suggests many benefits. Several aspects must be considered for future application of a LEA BDS in clinical facilities. The parameters of the magnets and the configuration of the optics design determine both the costs of the BDS and characteristics of the beam. This introduces a trade-off between the complexity and technical constraints imposed on the design and the achievable acceptance range: there must be an optimal range for which there will be maximal benefit. For example, Nesteruk et al. (113) describes that a ±30% energy acceptance band can provide ~70% of patient treatments at PSI without the energy modulation requiring a setting change. The design and optimisation process will likely be driven by this requirement which will outline the cost benefit, particularly for the delivery of other particle types and heavier ions.

This also raises the question of the appropriate source-to-axis distance and positioning of scanning magnets either upstream or downstream (17). What is clear is that in a novel BDS the parameters of the delivered beam must be clinically acceptable: energy spread (relates to range and beam penumbra), quality, size and shape (reproducible for every energy), position (spots must be positioned within precise margins) and also transmission (relates to particle rate for IES scans and current regulation with off-momentum particles). These properties must be consistent across the entire energy range and conform to performance and safety standards: rapid and accurate delivery cannot impinge on patient safety. Additional components (ripple filter, scattering foils etc.) may be necessary to moderate several beam characteristics upstream of the BDS (13, 120).

The build of any BDS must be as robust as existing commercial systems (mechanically and operationally) and accommodate all the necessary components (beam diagnostics, nozzle, ESS etc.). For integration, the BDS needs to consider modularity for possible retrofitting or replacement of parts. Fundamentally, one must expect a lighter or smaller physical structure, also a simpler system in terms of functionality, servicing and tuning; these improvements along with cheaper running costs will further assist to lower overall expense.

The adaptation of current control systems to manage a larger energy range is currently being explored. A recent study at PSI by Fattori et al. (121) demonstrates the clinical possibilities enabled by an increased momentum band to deliver PBS with real time tracking and enhanced rescanning capabilities. The prospect of energy meandering – ramping beamline magnets bidirectionally (up and down) – to further decrease the BDT is also presented. This combined with optimization of the energy sequencing and layering offers higher flexibility and uptime in terms of the duty cycle (93, 122).

### 3.4 Motion and Treatment Efficacy

The discussed benefits of decreased BDT have so far centered around the gain in delivery efficiency and therefore treatment efficiency or cost. Arguably however, the more compelling argument of a faster BDT is the potential of better treatment efficacy: treatment quality can be correlated to the efficiency of delivery (27). Future CPT facilities will need to be able to operate with shorter BDTs whilst ideally providing better quality treatments. As the BDT is dominated by the ELST, the accumulation of delays for each IES results in extended irradiation times; scanning sequences within 3–5 s or longer correspond with the respiration cycle and the effects of this motion are consequential for treatment (123).

#### 3.4.1 Interplay Effects

For PBS in CPT, there is an inherent challenge of utilizing the BP due to uncertainties in the range and physiologic motion which compromise any dosimetric advantages. Heavy ions have regions of elevated LET and therefore greater sensitivity, this makes it more challenging to treat a wide range of indications, especially for moving tumors. The issue of motion during PBS delivery is twofold: both the target site and the beam deviate in position simultaneously, resulting in degraded dose distributions, ‘*extitinterplay effects*’. Effects differ as dependent on the accelerator type, BDS and dose delivery characteristics (124).

**Figure 10:**
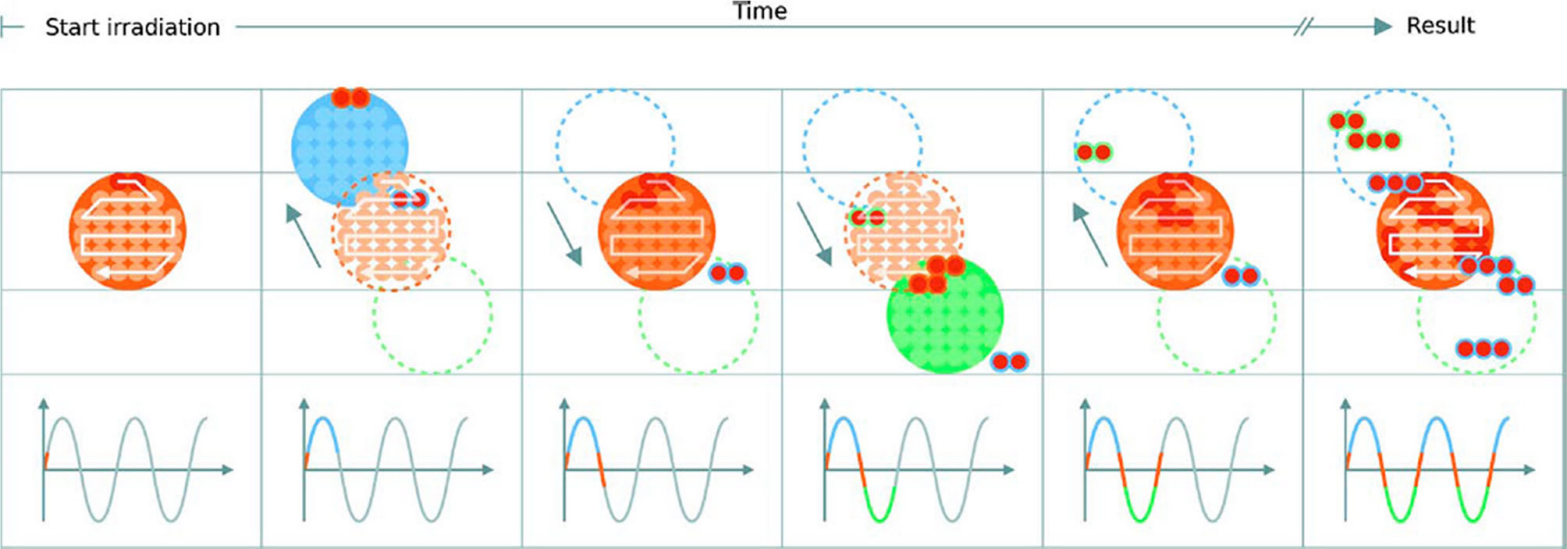
Delivery of a single IES with target motion (phases of movement are indicated by the plot and shown in blue, orange and green). The initially determined scan path in the target volume is shown in red. The raster scanned spots are translated outside the target due to motion which results in progressive degradation of the dose distribution. Reproduced from (124) with permission from IOP.

Interplay effects (**Figure 10**) cause regional dose in homogeneities due to under- and over-dosage, resulting in differences from the planned treatment distributions in each fraction. The clinical implications of interplay effects are well known (125–127) and require a variety of motion mitigation strategies; many are commonly used in practice and more are also being developed. It is frequently recommended that a shorter BDT can decrease the extent of or even prevent interplay effects, if the BDS is capable of delivering the dose sufficiently fast. The overall length of treatment is important: shorter irradiation times are ideal to reduce the amount of intrafractional motion yet to correct for interplay effects, more fractions are beneficial. It may seem like these are in conflict as each occur at the detriment of the other however, the key factor is again the BDT itself: high dosimetric quality has been demonstrated to be achievable even with higher delivery efficiency (27).

#### 3.4.2 Management & Mitigation

A shorter BDT is attainable by reducing the burden of long ELSTs: the longer the duration, the greater the need to minimize its impact. Work by Van De Water et al. (94) investigated the effect of a shorter BDT on plan quality by using a self-developed method with their treatment planning system to minimize number of layers required to deliver a treatment with robust optimization. It was shown that the BDT could be reduced by up to 40% for a range of different disease sites without compromising treatment quality. Other methods to decrease the BDT include: increasing the IES spacing (128), varying the size of spots (129), using a range of non-uniform sizes (13), changing the dose grid size or spotspacing (130), optimizing spot sequencing (131), scan path (132), or multiple criteria i.e. different weighted spots or resampling for selective placement of spots (27, 133).

Cao et al. (134) also present an energy layer optimization method which increased the delivery efficiency whilst maintaining dosimetric quality. Each of these has varying effects on dosimetric metrics such as homogeneity, conformity indices or equivalent uniform dose. However, some associated benefits are not quantifiable, such as patient comfort and further biological effects which may also contribute to better treatment outcomes. The purpose of any motion mitigation approach is to preserve conformity but simultaneously maintain treatment time duration (124). All of these corrective optimization tools are designed to work around existing limitations in technology and if a new, faster BDS and accelerator system were made available, would either become obsolete, or could be made even more powerful to the benefit of both treatment efficiency and efficacy.

There are also a range of common techniques which have been translated from XRT to CPT, including 4D planning and delivery (135–137); a comprehensive overview is presented by Bertholet et al. (138). A simple method is to implement safety margins in treatment planning, expanding the clinical target volume to a planning target volume to account for uncertainties and dose delivery errors (139). However, this has been demonstrated to be insufficient for complex intensity modulated PBT plans (140) and more robust methods are necessary to lessen adverse effects caused by the steep dose gradients and motion. Managing these is a highly complex task and a variety of motion mitigation strategies are applied by different facilities; these are summarized in detail in (141–143). Some specific approaches include: breath-hold (144), beam tracking (33), gating (145) which can also be combined with rescanning (31, 146). The use of physical equipment to shape the beam has also been re-examined using ridge filters (147, 148), 3D modulators (149) and other beam shaping (150) or modulating devices (151). Equivalent to passive scattering, the entire field can then be delivered almost instantaneously which thus negates the effects of interplay (1).

#### 3.4.3 Rescanning

In addition to beam gating and tracking, rescanning – also termed repainting (152) – is a primary method used to mitigate intrafractional motion through repeated irradiation. Pencil beams are particularly sensitive to motion and as this movement is generally periodic, dose errors can be statistically averaged out by increasing the number of fractions (153). A minimum number of rescans must be performed for added benefit (154), particularly for mobile sites such as the liver and lungs (127). Notably, the effect itself depends on the patient and beam parameters such as the direction, scan speed and path: characteristics determined by the accelerator and BDS. Bert et al. (124) mention that by choosing favorable parameters, the severity of interplay effects can be lowered and quasi-eliminated if scan speeds are sufficiently quick. The significant concern with rescanning and other mitigation techniques is that they can extend treatment to unacceptable lengths of time. Even at facilities which offer fast dynamic energy modulation, the accumulation of BDT still surpass time limits defined by the respiration cycle. The potential benefit of a faster BDS is the higher rescanning ability: this is specifically dependent on capabilities of the BDS, primarily its efficiency and the applied methods of delivery (29).

**Figure 11:**
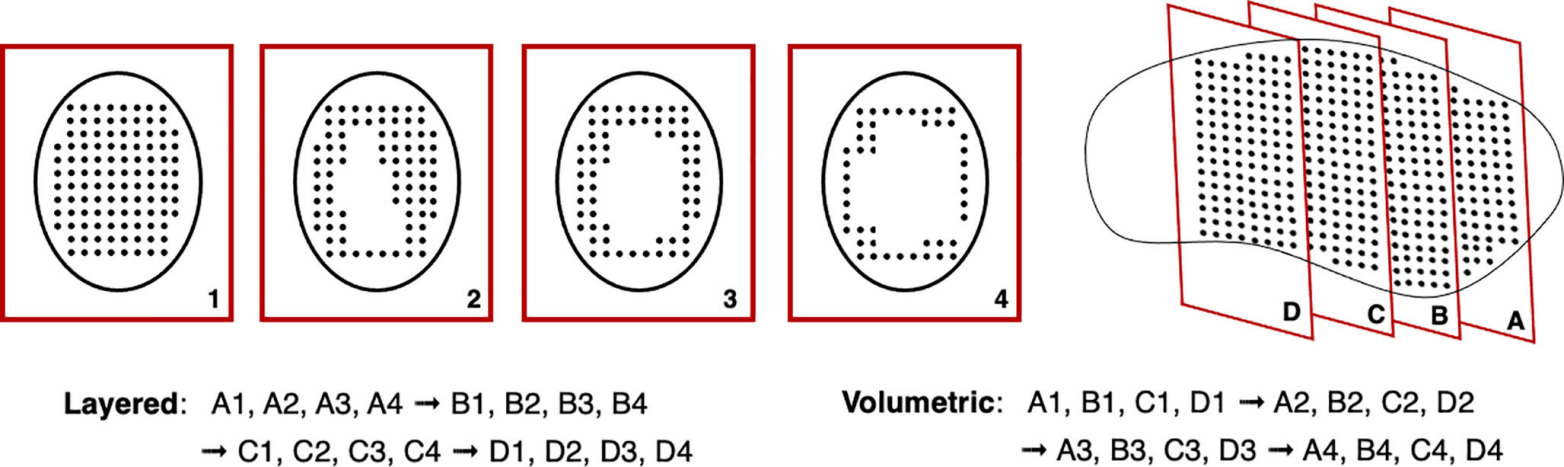
Possible example IES pattern sequences for layered and volumetric rescanning.

Another issue with rescanning is if motion of the beam and patient are synchronized: this jeopardizes the averaging effect. This can be avoided by ensuring delivery across the entire respiration cycle (i.e. phase controlled rescanning or breath-sampled rescanning) or introducing variations in the scan path by delays or randomness (142). There are several different patterns by which rescanning is performed (**Figure 11**), most commonly it is done akin to typical delivery, by painting repeatedly across an IES before moving onto the next consecutive layer (layered rescanning). An alternative method is to move through the different layers first, returning to the same IES to paint subsequent distributions (volumetric rescanning).

Volumetric rescanning (VR) is not employed clinically due to long ELSTs which make it impractical. Studies suggest several benefits as it enables additional scan paths and can alter the temporal correlation between beam and organ motion (141). Modifying the rescanning pattern to break the coherence of the beam structure with the periods of motion is an indicator of effectiveness; Bernatowicz et al. (155) demonstrated in a comparative study that outcomes may be less dependent on when the irradiation occurs during the respiratory phase, if VR is performed. The magnitude of the motion amplitude and duty cycle also impact the effectiveness, which can be machine specific (156); VR appears more sensitive to motion irregularity however this is likely due to extended ELST and treatment times (157). A study by Zenklusen et al. (152) suggests that combining VR with a fast delivery technique such as continuous line scanning can be an attractive method if it is possible to irradiate the entire volume within a single breath hold.

## 4 Emerging Applications

The field of CPT is evolving rapidly and the limitations of even state-of-the-art technology are becoming apparent; the possibility of volumetric rescanning and other advanced techniques require the BDS to be able to deliver efficiently with fast energy modulation. A recent review by Mazal et al. (109) outline several of these proposed CPT approaches to reduce associated uncertainties, complexities and cost. We specifically examine technological constraints and discuss BDS improvements as relevant for FLASH and arc therapy.

### 4.1 FLASH

The goal for treatment is to be able to irradiate the tumor sufficiently while sparing healthy tissue. This is represented by the therapeutic index (TI) and indicates the ratio between the probability of tumor control to normal tissue complication: improvements in delivery methodologies and treatment efficacy seek to increase the TI. There is always a trade-off with increasing the amount of dose delivered to the tumor, as normal tissue is simultaneously exposed to damaging radiation. Hyperfractionation and different approaches are commonly used in RT to vary the length of treatments to reduce toxicity and support the recovery of healthy tissue. Alternatively, some radioresistant tumors also respond well with hypofractionation. It is well established that the dose rate and irradiation time has an effect on cell response (158, 159) although it varies widely, dependent on biological parameters and the linear energy transfer (LET) related to the particle type (160). For certain conditions, a minimal dose rate effect has been observed; this has prompted a surge of recent research activity to reconsider applicable irradiation time scales for better therapeutic outcomes.

As such, the shift to ultra high ‘*FLASH*’ dose rates (≥40 Gy/s in ~100 ms) (161) has gained significant interest and may have the potential to revolutionise RT. The promise of FLASH therapy suggests an increased TI due to biological advantages by a reduction of normal tissue complications *via* the tissue sparing FLASH effect (162). Although the effect is dependent on spatio-temporal factors, the provision of FLASH RT may favor delivery with certain particle types based on technological compatibility and applicable physical parameters. However, the specific biological mechanisms are complex and still yet to be clearly identified (163–165). These drive the technical requirements necessary to induce the benefits and achieve clinical feasibility: the ‘*beam parameter space*’ determines the applicable radiation conditions such as the beam structure and particle type, yet much remains under investigation (166, 167).

The necessary accelerator and beam delivery developments required to deliver FLASH with clinical protons are detailed extensively by Jolly et al. (42). Alongside this is also the need for better instrumentation systems which can operate proficiently under FLASH conditions (168). A fundamental challenge is achieving the requisite FLASH beam parameters for PBS delivery with clinical accelerator systems, given safety restrictions (169). The generated beam intensity must be sufficiently high to realize the minimum effective FLASH dose rate and simultaneously, provide adequate coverage and conformity over the applicable fields. It has been easier to modify existing clinical LINACs to deliver FLASH with electron beams (170). For ion beams there are difficulties with reaching the required dose rates, which demand an increase in beam current by several orders of magnitude for rapid irradiation of a clinically relevant volume. A number of CPT facilities have been able to modify their accelerators (mostly isochronous cyclotrons and synchrocyclotrons) for FLASH with proton beams (171), and photon beams have been studied at large scale synchrotron research facilities (172). FLASH with different ions such as carbon and helium is also being examined (173–175).

For conventional synchrotrons, one of the challenges is to be able to store enough particles in the main ring to deliver the entire field, as the time required for a single re-injection and acceleration is typically considerably longer than 500 ms. Systems theoretically capable of injecting into the main ring at a suitable energy with a charge exceeding requirements do exist (78). However, developments in this direction are in opposition to the goal of footprint and cost reduction, as larger and more expensive equipment is generally necessary. When a large amount of particles is injected in the main ring at once, the interaction among the particles start to be less and less negligible. Although strategies exist to keep this space charge effect under control, the effect is accentuated in smaller radius synchrotrons.

A study by Zou et al. (176) assessed the limitations experienced with cyclotrons by analyzing the main machine parameters which influence delivery. The authors demonstrate that it is impossible to deliver FLASH dose rates fully across the planned 5 × 5 × 5 × cm^3^ SOBP region due to BDS dead times: magnet scanning speeds and significantly, the ELST. Nine IES scans were required and although applying a standard 1.5 s ELST was too slow, even the fastest clinical ELSTs of 50 ms and 80 ms, were also insufficient. High doses were achieved in the central beam spot axis but at the lateral edges, a significant portion fell below nominal levels. This spread of dose across the beam spot is a noted difficulty with delivering FLASH using PBS however the necessary instantaneous, mean or threshold dose over a region of interest, has not yet been quantitatively defined (177). The impact of deadtimes is also unclear, as PBS parameters (components in Section 2.2) determine the dose output and timing (178). Hybrid delivery schemes and 3D modulation devices have been suggested for reaching FLASH dose rates across the entire volume however higher beam intensities are needed to compensate particle losses.

Near instantaneous delivery should be targeted given the indicative 100 ms time frame necessary for the FLASH effect: this will also negate the effects of intrafractional motion. This rapid delivery of FLASH combined with image guidance is also being developed into a next generation, treatment modality: Pluridirectional High-energy Agile Scanning Electronic Radiotherapy (PHASER) (179). The platform consists of a novel high gradient LINAC structure, distributed RF network for 16 non-coplanar beamlines in place of a gantry, where the electron beam is steered onto the X-ray source and collimated into fine channels. This could enable the delivery of high intensity, modulated XRT beams from multiple angles with fast energy changes (300 ns) for FLASH.

Clinical FLASH trials have also commenced (180, 181) yet there is limited implementation due to many challenges: the technological requirements push the boundaries of and surpass current capabilities. Multiple experimental setups have been developed (182–185) investigating the applicability of these adaptations at clinical PBT facilities. Passive scattering systems with cyclotrons can deliver a SOBP with sufficient mean dose rates but face difficulties reaching larger fields given transmission losses. Improvements are needed for depth and lateral modulation: it is still unclear what optimal beam parameters (time structure, profile, range, uniformity, field size etc.) will be feasible in practice (167).

Furthermore, as ensuring precise beam delivery and positioning is difficult, transmission or ‘*shoot-through*’ FLASH (186) with protons is performed where exploitation of the BP may be considered redundant to maintain a high, effective mean dose rate whilst also resulting in the FLASH effect (184). Several transmission studies have been reported (177, 178, 187) as this method is achievable with current technologies. This bypasses the need for additional beam modification devices, minimizing range uncertainties and delivery requirements. However, increased tissue sparing, dose conformity and other clinical benefits require use of the BP alongside multiple fields of different energies (188). There may also be further radiobiological advantages but this and the FLASH induced responses specific to different radiation types are still being explored.

### 4.2 Arc Therapy

The endeavor to speed up treatment times has recently renewed interest in arc therapy which is already a mainstream modality in XRT (i.e. Volumetric Modulated Arc Therapy, VMAT). Radiation is delivered to the patient as the gantry rotates rather than with multiple fields of differently angled, fixed beams. It is possible to achieve higher quality XRT plans with VMAT and a significantly faster BDT than with multiple static beams (189). This concept applied to PBT is termed proton arc therapy (PAT) (190), combined with PBS, spot-scanning proton arc (SPArc) (191) and with other ions (helium and carbon), spot-scanning hadron arc (SHarc) therapy (192). This delivery technique is highly complex and a fundamental challenge again lies with the capacity of the BDS: it must deliver reliably and continuously along rotational arcs, with the ability to switch quickly between energy layers (193).

**Figure 12:**
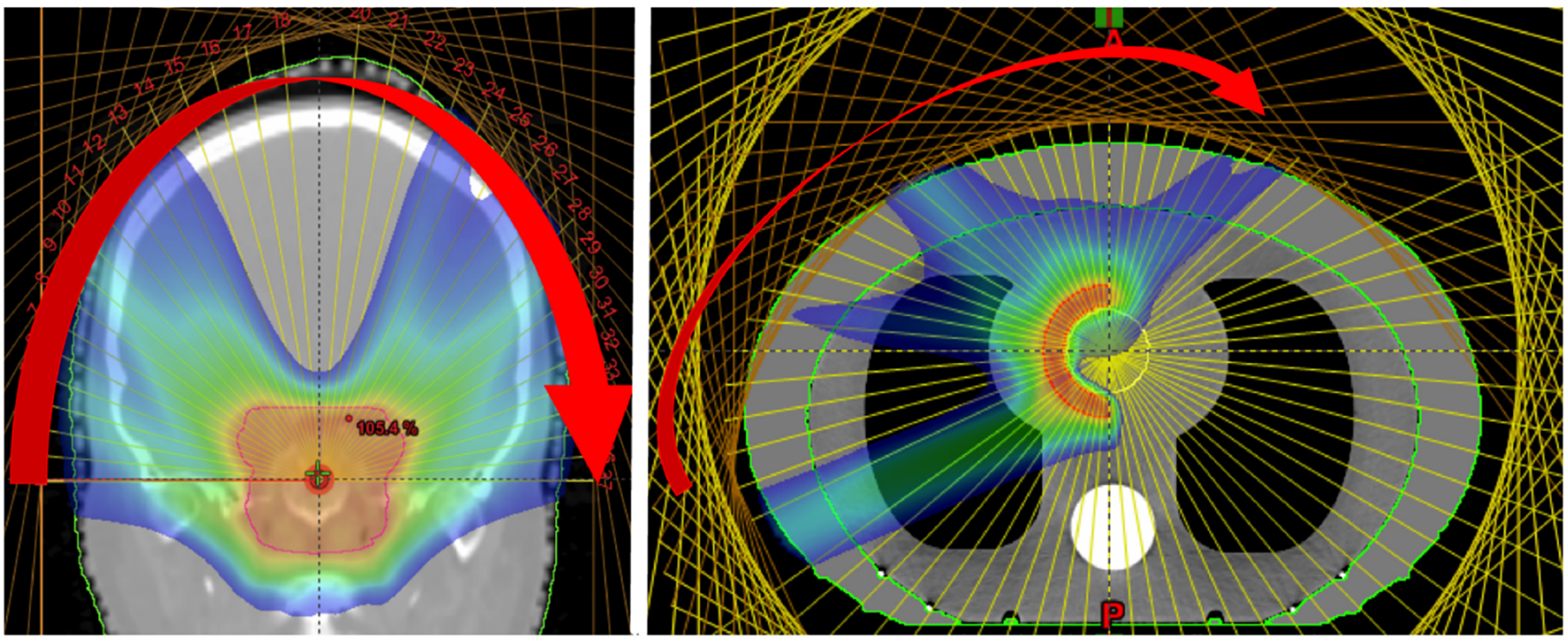
Dose distributions obtained from the delivery of mono-energetic PAT. Coronal view of 37 fields for a brain tumor treatment, applied by a couch rotation every 5° (left). Axial view of a tissue equivalent lung phantom using 35 fields, also 5° rotation (right). Reproduced from (194) with permission from IOP.

In PAT (**Figure 12**), a spot scanned beam is delivered in a continuous arc which effectively dilutes the impact of range uncertainties, achieving a conformal dose distribution with also a reduction in standard entrance dose (195). This allows greater flexibility in positioning high dose regions along the beam path and the potential for a much shorter BDT (15). Ding et al. (196) have shown that good conformity and the possibility of a lower integral dose could be achieved with SParc however, treatment plans must be optimized for robustness and efficiency (194, 197, 198). Significant savings in BDT were reported (191) with continuous arc delivery however this is not yet clinically possible with current technology, due to complexities with gantry rotation and long ELSTs.

As an alternative, conventional (step-and-shoot) spot delivery was suggested with a moving couch for fixed beamlines as well as better timing synchronization. Carabe-Fernandez et al. (193) emphasized that although more investigation is needed, PAT has potential particularly for certain indications (brain tumors). Once again, in PAT the delivery efficiency is determined by the BDS and improvements are required such as better stability with beam current, positioning and fast energy switching. Single energy fields can reduce BDT such as in proton monoenergetic arc therapy (PMAT) (194), showing acceptable coverage and plan quality. However, partial arcs of varying energies have been proposed for better biological (LET) optimization with complex geometries (199). As the total BDT is a limiting factor (195), quicker ELSTs can shorten treatment times which will also lessen dosimetric constraints due to a dependence on single fields.

**Figure 13:**
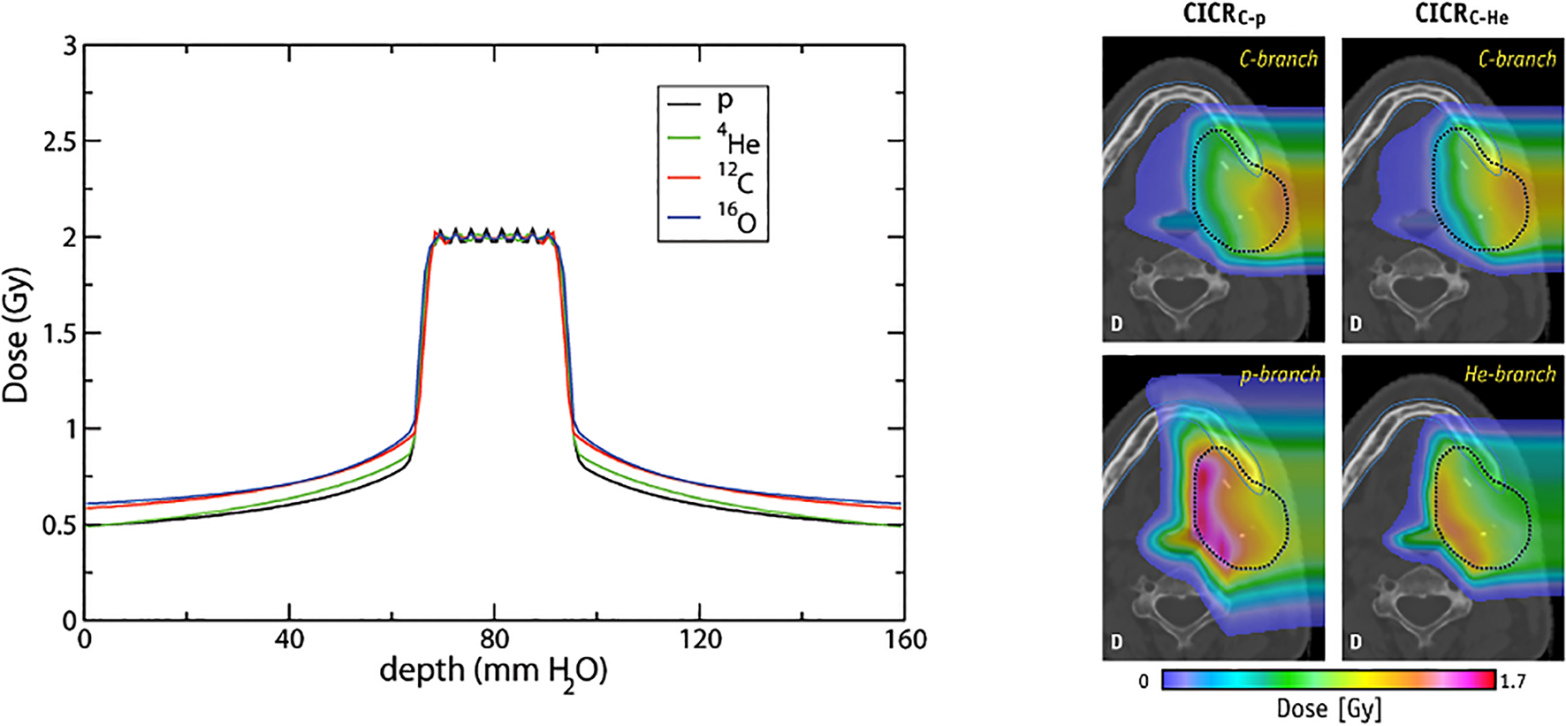
Conformal distributions can be produced by applying opposed fields using different particle types, optimizing for physical dose (left) (201). Combining beams of multiple particle types can generate distinctly different dose distributions, by positioning a chosen particle branch (p or He) at the distal end of the field (202). Images distributed under CC BY 4.0, BY-NC-ND.

Additionally, the possibility of an increased dose in the target volume with PAT may not translate to a higher degree of conformity however could exploit radiobiological advantages further by increasing the TI (199, 200). As with multi-ion radiotherapy (MIRT), combining different particle types for an effective mix of low- and high-LET regions could generate a higher dosimetric quality plan by utilizing favorable characteristics. A planning study by Mein et al. (192) evaluates SHarc with different field configurations using proton, helium and carbon ion beams (**Figure 13**). The results demonstrate several possible clinical benefits such as a lower dose bath, minimization of high-LET components on critical structures and better tumor control with normal tissue toxicity reduction. The use of multiple beam energies offer further gain over single or two-field plans however there are clear, unresolved technical hurdles with the present BDS and gantry systems which prevent the actualization of this technique.

## 5 Discussion and Future Directions

It is contended that CPT will be a widely adopted modality once costs are comparable to XRT. Global prevalence is growing and new and exciting advancements are on the horizon: delivery methodologies, novel design concepts and greater biological advantages. The current landscape encourages exploration of future long- and intermediate-term approaches which will allow CPT to exceed the ceiling achieved by state-of-the-art XRT (15). Evidently, next generation technologies and facilities are needed to address present challenges in CPT.

Several innovative concepts of delivery have emerged which offer hope of full exploitation of the unique advantages offered by CPT. However, among the multitude of prospective improvements for beam delivery, there is a common underlying goal, to address challenges surrounding treatment time. This is complex as it encompasses more than the BDS and technology, as was examined in Section 2. A shorter BDT results in not only a shorter treatment time, but is also consequential in terms of costs and treatment quality. The constraint imposed by the long ELST is a distinct hurdle in minimizing the BDT: alleviating this would result in better treatment efficiency by reducing involved costs, increasing throughput, also improving treatment efficacy.

Gantries account for the largest expense of facilities and more compact systems have been designed for CPT however the general ambition has been mostly for size and cost reduction. Fundamentally, these hinge on the optical design and the parameters of the magnets, new possibilities are becoming feasible with SC technology however limitations and issues with ramping speeds still persist. Nevertheless, an alternative approach is to improve the beam transport capabilities of the BDS and redesign the optics to increase the overall momentum acceptance range; this has the potential to have a significant impact on treatment by eliminating the ELST dependency on technological bounds, thus shrinking the BDT. The feasibility of a LEA BDS as a solution to decrease the BDT is discussed in Section 3.3.1. The prospect of a LEA BDS raises several challenges but has the possibility of achieving higher quality treatments at lower incurred costs, however would need to be supplemented by further technological improvements throughout. Recent developments with accelerators and possibilities to further decrease the BDT are discussed in Section 3.1.

The ELST handicap on BDT is almost entirely dependent on technological limits: a myriad of different methods must be used in the clinic in order to provide effective treatments to circumvent existing capabilities. These add onto the delivery scheme and workflow; treatment planning optimization is required or the BDS must be adapted to directly change the beam energy using mechanical components. This includes various approaches outside of the BDS such as patient specific devices as well as different accelerator feedback and extraction schemes. A shorter BDT has a significant clinical benefit and the impact of motion and interplay effects must be mitigated using various strategies, described in Section 3.4. Many of the mentioned approaches could also effectively reduce BDT and improve conformity if implemented during the planning optimization process in general clinical practice (13).

A faster BDT and energy switching also drives developments toward a future BDS capable of delivering treatments for a wider range of indications, also with advanced techniques. Several emerging applications are anticipated in the near future which will require an improved BDS for successful delivery such as FLASH and arc therapy. Several other anticipated developments in CPT are not discussed in detail but are also mentioned for context. Faster irradiation times go hand in hand with the need to ensure that treatments are still delivered with the necessary requirements of safety and precision. The importance of robust planning is also arguably higher for CPT than XRT but more challenging due to the physical uncertainties, geometrical in homogeneities and inter- and intra-fractional motion. This is again significant when considering different particle types. CIBT is expanding in addition to MIRT possibilities using beams of helium, oxygen, lithium etc. (201, 203) which could further increase treatment efficacy. The combination of different ions offers a realm of new possibilities, by tailoring the desired LET and radiobiological attributes for different cancer sites. Optimization of these dose regions can offer more stable distributions and effective treatments (**Figure 13**), such as using lower-LET particles for the sharper dose fall-off and higher-LET beams for hypoxic or radioresistant tumors (204).

MIRT is still a developing modality and technical limitations are primarily due to difficulties with the ion sources and long switching times. Considerations are also necessary with the acceleration and beam transport, as it is more difficult to deliver beams of heavier ions given the associated beam dynamics, mechanical and physical requirements (205). The accelerator complex and BDS will need to be able to accommodate the range of different particle types; this may be selective based on characteristics such as mass and charge [i.e. mass-to-charge ratio ≤3 (206)]. For MIRT treatments, the beams are expected to be arranged such that the high dose regions fall appropriately within the target volume, hence this corresponds to different beam energies for each individual particle type.

The method of delivery must also be gauged, techniques such as minibeams and spatially fractionated RT are suggested (6). Moreover, as the ion switching and ELST restrictions currently cause long BDTs, MIRT treatments have firstly been studied in a single field arrangement (202) and opposed fields (63). The clinical flexibility and advantages are demonstrated however it is worth to note that technological possibilities may only allow sequential irradiation: this raises questions about throughput, quality assurance requirements, interplay effects, motion mitigation and fractionation schedules. The unknowns with the biological effects are also crucial, aside from the uncertainties with modelling and determination of treatment outcomes, the irradiation time structure and the division of the BDT between sources and fractions may introduce considerations with radiobiological chronicity (204).

Simultaneous delivery with mixed beams is complex however has been performed for online monitoring and range verification with helium and carbon (206, 207), exploiting the difference between BPs: carbon ions were used for treatment and helium for simultaneous imaging. Mixed fields are practically limited to synchrotron facilities as cyclotrons aren’t able to achieve the acceleration requirements for heavier ions at clinical treatment depths, also the presence of an ESS changes the particle energy and velocity ratio (208). Nonetheless, the strengths of MIRT and fundamentally CPT, can be achieved when limiting factors with delivery and motion are resolved; another important element with this is the need for precision imaging (209).

Online, volumetric imaging for (also adaptive) treatment planning, continuous patient monitoring, motion compensation and 4D treatment delivery is not yet readily clinically available for CPT; the value of these however have a higher potential for benefit in comparison with XRT (210). A promising avenue for this is with MRI guided PBT (MRPT), which has the capability of providing fast real-time imaging with superior soft-tissue contrast without the drawback of additional radiation exposure (211). This approach is also still being developed however there are several complex challenges with integrating MRI technology with a PBT system. The influence of the MRI magnetic field affects the trajectory of the proton beam, interfering with both the delivered dose distribution and resulting image quality. Corrections are required to compensate for the beam deflection and deviations in the treatment plan, dependent on the MR magnet field strength (212, 213). The associated technological concerns relate to beam delivery and decoupling the PBS beamline from the MRI magnet: this requires an entirely new BDS design which can accommodate the physical and geometrical aspects of both systems (214–216). The use of multi-modal approaches for enhanced imaging is also of interest and combining i.e. CT with MRPT has exhibited benefits (13, 217).

## 6 Concluding Remarks

The primary hurdle with CPT remains a question of cost, its availability and accessibility is still driven by the balance between cost and benefit: progressive improvements will contribute to decreasing the cost however future growth will depend on the extent of benefit (24). This is also influenced by various factors such as patient selection, clinical trials and scientific evidence. CPT still favors shorter treatments as it is difficult to immobilize patients for larger or complex lesions requiring extended treatment times (i.e. >30 mins). Increasing the range of accepted indications for treatment and capitalising on biological benefits (i.e. reducing fractions) supports the pursuit of reaching the same cost-effective levels as XRT (218, 219).

However, there are several challenges which impact the delivery efficiency and efficacy of treatments in CPT. We have reviewed the existing technical limitations related to the BDS and accelerator, identifying potential avenues for development in CPT. Focusing on the BDS, enhancements such as a LEA could reduce the limiting impact of the ELST on the BDT and shorten treatment times. This supports potential benefits such as cost reduction by expanding the utility of CPT and increasing the throughput of faster and higher quality treatments. Fast energy variation would also offer the capability of delivering advanced methodologies such as volumetric rescanning, FLASH and arc therapy. Improvements in beam delivery and related technologies enable the possibility of a future with cheaper, faster, precise and more effective CPT treatments.

## Author Contributions

JY and SS: conception and design of the study. JY: literature review, manuscript preparation, writing, editing and figures. AF: writing and review. SS: writing, review, editing and figures. All authors contributed to the article and approved the submitted version.

## Funding

This work was supported by the William Stone Trust Fund and the Laby Foundation.

## Conflict of Interest

The authors declare that the research was conducted in the absence of any commercial or financial relationships that could be construed as a potential conflict of interest.

## Publisher’s Note

All claims expressed in this article are solely those of the authors and do not necessarily represent those of their affiliated organizations, or those of the publisher, the editors and the reviewers. Any product that may be evaluated in this article, or claim that may be made by its manufacturer, is not guaranteed or endorsed by the publisher.

## Footnotes

1 Existing recommendations and requirements for technological improvements have been primarily focused on PBT, which precede developments for CPT with heavier ions due to greater clinical experience. We include many references to PBT for readers to explore these where relevant.

2 The energy change is fixed to a single direction.

3 In this scenario the re-injection times are also not improved from the typical operation mode.

4 Another arguably more elegant method is the use of a rotator (55) which physically rotates a set of magnets to match the optics for different gantry angles, requiring no additional time for energy switching (apart from the magnetic field setting in the BTL).

5 Although designs for cyclotrons dedicated to particle therapy with He or C exist (64, 65), no facility has so far been developed yet.

6 An extreme example is LIPAc (78) which has already demonstrated the acceleration of 5 MeV, 125 mA deuteron beam which is ˜50 times more than the currents typically injected in synchrotrons for therapy.

7 FFA is a method of designing accelerator optics and can be applied also to beamlines and gantries.

8 Slow extraction may be possible, but has not been studied in detail.

9 Simple additive estimation where *N* is the number of IES, BDT = (N)**T_1_** +(N-1)**T_2_** +(N)**T_3_**.
